# Into the weeds: Matching importation history to genetic consequences and pathways in two widely used biological control agents

**DOI:** 10.1111/eva.12755

**Published:** 2019-01-04

**Authors:** Julie V. Hopper, Kent F. McCue, Paul D. Pratt, Pierre Duchesne, Edwin D. Grosholz, Ruth A. Hufbauer

**Affiliations:** ^1^ Department of Biological Sciences University of Southern California Los Angeles California; ^2^ Crop Improvement and Genetics Research Unit USDA/ARS Albany California; ^3^ Invasive Species and Pollinator Health Research Unit USDA/ARS Albany California; ^4^ Département de Biologie Université Laval Québec City Québec Canada; ^5^ Department of Environmental Science and Policy University of California, Davis Davis California; ^6^ Department of Bioagricultural Science and Pest Management Colorado State University Fort Collins Colorado

**Keywords:** adaptation, biotype, classical biological control, herbivore, invasive, population genetics

## Abstract

The intentional introduction of exotic species through classical biological control programs provides unique opportunities to examine the consequences of population movement and ecological processes for the genetic diversity and population structure of introduced species. The weevils *Neochetina bruchi* and *N. eichhorniae* (Coleoptera: Curculionidae) have been introduced globally to control the invasive floating aquatic weed, *Eichhornia crassipes*, with variable outcomes. Here, we use the importation history and data from polymorphic microsatellite markers to examine the effects of introduction processes on population genetic diversity and structure. We report the first confirmation of hybridization between these species, which could have important consequences for the biological control program. For both species, there were more rare alleles in weevils from the native range than in weevils from the introduced range. *N. eichhorniae *also had higher allelic richness in the native range than in the introduced range. Neither the number of individuals initially introduced nor the number of introduction steps appeared to consistently affect genetic diversity. We found evidence of genetic drift, inbreeding, and admixture in several populations as well as significant population structure. Analyses estimated two populations and 11 sub‐clusters for *N. bruchi *and four populations and 23 sub‐clusters for *N. eichhorniae*, indicating divergence of populations during and after introduction. Genetic differentiation and allocation of introduced populations to source populations generally supported the documented importation history and clarified pathways in cases where multiple introductions occurred. In populations with multiple introductions, genetic admixture may have buffered against the negative effects of serial bottlenecks on genetic diversity. The genetic data combined with the introduction history from this biological control study system provide insight on the accuracy of predicting introduction pathways from genetic data and the consequences of these pathways for the genetic variation and structure of introduced species.

## 1 INTRODUCTION

In the modern era of global trade, species are being inadvertently and deliberately introduced widely beyond their historic ranges (Crowl, Crist, Parmenter, Belovsky, & Lugo, 2008; Lockwood, Hoopes, & Marchetti, 2013; Mack et al., 2000). A crucial focus of evolutionary ecology of introduced species is to understand their pattern of spread and to identify their native origins and pathways of introduction to better prevent and manage biological invasions (Estoup & Guillemaud, 2010). Inferring the origins and spread of these exotic species is challenging and rarely are the true pathways or origins known. Thus, a fruitful approach may be to use documented introductions, such as those performed in classical biological control, as model systems to provide greater insights into population genetic analyses, as well as insight into the consequences of population movement and ecological processes for the genetic structure and variation of a species (Fauvergue, Vercken, Malausa, & Hufbauer, 2012; Marsico et al., 2010).

Classical biological control uses natural enemies (predators, parasitoids, and pathogens) to control invasive populations of weeds, and arthropod pests and disease vectors in the introduced range (Van Driesche, Hoddle, & Center, 2008). These natural enemies, as biological control agents, are often imported across disjunct geographic ranges for the long‐term control of the target invasive species. In the modern era, these importations are well‐regulated (Van Driesche et al., 2008) and well documented (but see Coulson, 1992; Marsico et al., 2010). Thus, they provide model systems to study the repercussions of invasion pathways and multiple introductions—including their effects on inter‐ and intraspecific hybridization, bottlenecks, inbreeding, genetic variation, and correlations of genetic diversity with population performance of the biological control agents (Fauvergue et al., 2012; Marsico et al., 2010; Roderick & Navajas, 2003).

To enhance the establishment and success of biological control agents, often multiple separate introductions are made, and large numbers of individuals are released (Van Driesche et al., 2008). Multiple introductions here refer to introducing individuals from more than one population, or of more than one species, or both into the same geographic areas. Multiple introductions can increase the genetic diversity in an introduced population due to genetic admixture of different source populations (Bock et al., 2015; Dlugosch, Anderson, Braasch, Cang, & Gillette, 2015; Dlugosch & Parker, 2008; Rius & Darling, 2014; Szucs, Eigenbrode, Schwarzlander, & Schaffner, 2012). Alternatively, multiple introductions of more than one population could interfere with local adaptation, particularly in the native range (Rius & Darling, 2014; Verhoeven, Macel, Wolfe, & Biere, 2011). Additionally, hybridization can occur when more than one closely related species or strain is introduced, which can potentially lead to hybrid breakdown or hybrid vigor (Andersen & Mills, 2016; Arcella, Perry, Feder, & Lodge, 2014; Bean et al., 2013; Bitume, Bean, Stahlke, & Hufbauer, 2017; Mathenge et al., 2010; Szűcs et al., 2018). Hybrid vigor can result from positive epistatic interactions among loci, heterosis due to masking of deleterious alleles, or heterozygote advantage, whereas hybrid breakdown can occur from negative epistatic effects among loci and/or the underdominance of loci (Arcella et al., 2014; Edmands, 1999). Thus, the presence of multiple introductions and hybrids can greatly impact the growth and spread of introduced populations, and the efficacy of biological control programs.

The introduction of large numbers of individuals is critical to improve establishment success, as it buffers against demographic stochasticity and helps minimize loss of genetic variation (Fauvergue et al., 2012; Fraimout et al., 2017; Simberloff, 2009). Nonetheless, introduced populations often endure demographic bottlenecks (Dlugosch et al., 2015; Dlugosch & Parker, 2008; Estoup et al., 2016), which can decrease allelic richness and heterozygosity, with the latter depending on the rate of population growth following the initial bottleneck (Bock et al., 2015; Fauvergue et al., 2012; Nei, Maruyama, & Chakraborty, 1975). Certain alleles might increase or decrease in frequency by chance during bottlenecks, leading introduced populations to diverge from native populations (Dlugosch et al., 2015; Dlugosch & Parker, 2008). Genetic drift and inbreeding can also lead to increased homozygosity (Crow, 2010), which can be associated with reduced fitness (Bock et al., 2015) (;but see Verhoeven et al., 2011). However, population bottlenecks do not always reduce genetic variation (Estoup et al., 2016; Goodnight, 1988; Kolbe et al., 2004; Taylor, Downie, & Paterson, 2011) or lead to genetic differentiation from the native population (Franks, Pratt, & Tsutsui, 2010), particularly if populations grow rapidly following introduction (Nei et al., 1975). Evaluating the effects of bottlenecks in population size on genetic diversity can enhance our understanding of the consequences of introductions and spread of species.

Although great efforts are taken to introduce many individuals from the native range to enhance establishment success, regulatory processes can make this difficult. Thus, the number of individuals (propagule size) imported to a region ranges widely from 10 to more than 1,000. While regulations vary by country (De Clercq, Mason, & Babendreier, 2011), each collection from the native range for release typically passes through quarantine to prevent unintentional introductions of other species (Hufbauer, Bogdanowicz, & Harrison, 2004). In many countries, such as the United States, further screening to characterize host range is often required for each new collection from the native range, which can mean many additional generations in quarantine even for agents that have already been approved. During this time, inbreeding and adaptation to the quarantine and mass‐rearing environment can also occur (Freitas, Morales‐Correa, Barbosa, & Fernandes, 2018; Hopper, Roush, & Powell, 1993; Hufbauer et al., 2004). Following quarantine screening, population size is typically increased as much as possible (“mass rearing”) in order to release hundreds to thousands of individuals (e.g., see importation history section in this study). However, the proportion of individuals that survive in the field and contribute to the next generation may be low, resulting in another demographic bottleneck (Hufbauer et al., 2004).

Regulatory and logistical obstacles limit sampling from the native range; thus, biological control agents for release in new regions are often collected from a population already in use for biological control rather than revisiting the native range. This introduction process is analogous to the movement of invasive species, whereby an introduced population becomes the source of several secondary introductions, and is therefore acknowledged as a “bridgehead population” (Bertelsmeier & Keller, 2018; Dittrich‐Schröder et al., 2018; Fraimout et al., 2017; Lombaert et al., 2010). Similarly, biological control agents frequently undergo serial importation steps, and thus serial bottlenecks in population size. By using the known introduction pathways from biological control programs, we can evaluate our ability to reproduce the introduction pathways by analyzing data from molecular markers.

Here, we examine the importation history, genetic diversity, and population structure of two closely related species introduced for biological control to gain insight into the consequences of population movement and ecological processes for the genetic structure and variation of these two species. Here, we ask: (1) Is there evidence of hybridization between these species, and (2) how do introduction processes affect the genetic variation and structure of these species? More specifically, (2a) are there indications of decreased heterozygosity and allelic diversity in the introduced populations relative to the native range, (2b) do increases in the number of individuals initially released or genetic admixture from multiple introductions result in increased genetic diversity, (2c) do populations with more introduction steps between them and the source population in the native range exhibit greater loss in genetic variation compared to populations with fewer introduction steps, and (2d) despite originating from the same initial populations, have introduced populations differentiated from the native range and from each other?

To address these questions, we use the documented importation history and polymorphic microsatellite loci of two weevils, *Neochetina bruchi *and *N. eichhorniae *Hustache (Coleoptera: Curculionidae) from their native and introduced ranges. These two weevils are the most widely used biological control agents of water hyacinth, *Eichhornia crassipes *(Hill, Coetzee, Julien, & Center, 2011)*,* a floating aquatic plant native to South America. Water hyacinth is recognized as one of the world's worst invasive weeds (Hopper et al., 2017; Spencer & Ksander, 2005). Classical biological control of water hyacinth has been implemented across the globe, with some introductions resulting in significant reduction in water hyacinth cover and/or biomass, including parts of Australia, China, East Africa, the U.S. Gulf Coast, India, Mexico (Aguilar, Camarena, Center, & Bojorquez, 2003; Akers, Bergmann, & Pitcairn, 2017), and the lower elevation regions of South Africa (Julien, Hill, Center, & Jianqing, 2000). Releases of *N. bruchi *and *N. eichhorniae* from the native range (South America) began in the early 1970s, with initial and subsequent releases in 30 and 32 countries, respectively. These weevils have contributed substantially to the control of water hyacinth in at least 13 countries (Julien et al., 2000).

**Figure 1:**
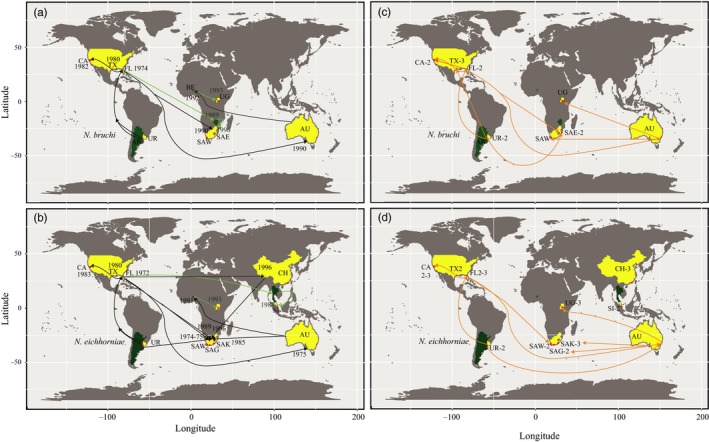
Partial importation history (a, b) compared to the introduction processes predicted by FLOCK analyses (c, d) of *Neochetina bruchi *and *Neochetina eichhorniae, *two weevils native to South America. Arrows depict the direction of the biological control releases and the date initially released, but do not point to the exact release site in that locality. Red markers are based on the GPS coordinates of the localities used in this study. Black lines and yellow‐filled regions represent the routes of importation history that were tested with microsatellite markers. Green‐filled regions and lines (routes) (arrows) were not tested with the genetic markers from this study, but represent relevant importation history to some of the tested regions. Abbreviations are detailed in Table 1. Numbers next to abbreviations indicate the number of sub‐clusters found from FLOCK analyses (c, d)

Through their use as biological control agents, these two weevil species have often undergone multiple and serial introductions (Figure 1). For example, in the United States, weevils of *N. eichhorniae *released into northern California underwent four sequential importation steps from the original Argentinian population in the native South American region. Native Argentinian weevils were released into USA: Florida in the 1970s, and the weevils in USA: Florida were used to found a population in USA: Louisiana, which were then used to found populations in USA: Texas. This USA: Texas population was the source for the northern California population released in the early 1980s (Stewart, Cofrancesco, & Bezark, 1988). Similarly, in South Africa, there were multiple introductions of each *N. bruchi *and *N. eichhorniae *with each new release being sourced from a different location to which they had been introduced for biological control (Cilliers, 1999), rather than directly from the native range. These multiple introductions from the non‐native range represent serial bottlenecks in population size that could potentially reduce genetic diversity and limit adaptive potential. Alternatively, these multiple introductions from different source populations could increase genetic diversity through genetic admixture of the different source populations (see Bock et al., 2015; Dlugosch et al., 2015; Dlugosch & Parker, 2008). The latter may occur particularly if each source population had sufficient time to diverge or adapt to the region of introduction, resulting in increased genetic differentiation from its source population.

Based on the importation history and documented releases of these two biological control agents, we proposed several hypotheses addressing our five study questions in turn. (1) We hypothesized that hybrids of these two species would occur, as they have frequently been co‐introduced to the same geographic regions (Julien et al., 2000) and individuals with morphological characteristics of both species have been found (Hopper et al., 2017). (2a) We hypothesized that genetic diversity (here as heterozygosity and allelic richness) would be the highest in the native range (Uruguay) compared to regions where the weevils were introduced as biological control agents. Similarly, we hypothesized that the populations from the native range would have more rare (private) alleles than the introduced regions. (2b) We hypothesized that allelic richness would reflect the number of individuals released (propagule size). Specifically, as the initial propagule size of *N. bruchi *was greater than that of *N. eichhorniae *in USA: Florida, we expected that populations of *N. bruchi *in USA: Florida would be less likely than populations of *N. eichhorniae* to lose rare alleles and exhibit reduced allelic richness in the introduced range compared to the native range. In cases where multiple introductions were prominent, particularly regarding the introduced populations of *N. eichhorniae *in South Africa, we hypothesized that the genetic admixture would increase genetic diversity and buffer against the negative effects of serial bottlenecks. (2c) On a global scale, regarding the number of serial introductions, we hypothesized that populations with more introduction steps away from the native range would harbor lower genetic diversity than those with fewer steps. (2d) As the initial releases occurred over 40 years ago, we hypothesized that despite originating from the same initial populations that most introduced populations would have diverged genetically from the native range and each other.

## 2 MATERIALS AND METHODS

### 2.1 Relevant importation and release history of *N. bruchi *and* N. eichhorniae*

Importation and release history were obtained from peer‐reviewed literature, government reports (Cilliers, 1999; Confrancesco, 1981; Hendrich, Balke, & Yang, 2004; Julien et al., 2000; Manning, 1979; Stewart et al., 1988; Vanthielen et al., 1994; Winston et al., 2014), shipment letters (https://www.nal.usda.gov/), unpublished quarantine records (USDA, ARS Biological Control Laboratory, Gainesville, Florida), and published quarantine records (USDA ARS, 2018). However, there were many gaps, as details in the importation and release history of biological control agents are often missing or not easily accessible to the public (Coulson, 1992; Marsico et al., 2010) including the number of adults surviving shipments, the number used for mass‐rearing after quarantine inspection, the number ultimately released, the localities of the releases, and whether multiple releases occurred.

From the shipment letters and quarantine reports pertaining to the initial exports from Argentina to USA: Florida (prior to global dispersal), it appears that samples from at least two populations of *N. bruchi *and *N. eichhorniae *were collected from Argentina and released in USA: Florida. Initial shipments of *N. bruchi *received in 1974 to the USA: Florida quarantine consisted of 156 and 1,050 surviving adult weevils from collections in Campana Lagoon and Dique Lujan, Buenos Aires, Argentina, respectively. However, it is unclear whether or not individuals from Campana were used for mass rearing, based on notes about possible infections by nematodes. Additional shipments from these collection sites appear to have occurred around this same time, but it cannot be confirmed whether they were used for augmenting the populations that were eventually released. Samples from two populations of *N. eichhorniae* were collected and shipped in 1971, with the number of surviving adults arriving in the quarantine in USA: Florida documented as 10 from Campana Lagoon and 156 from Santa Fe, Argentina, with these collection sites *c.* 300 miles apart. An additional third population of *N. eichhorniae *may have been received, containing a mixture of 219 weevils from Campana and Dique Lujan Buenos Aires and arriving in 1975 (https://www.nal.usda.gov/). However, these reports indicated potential nematode and fungal infestation in this later shipment, and again it was not clear whether or not offspring from these weevils were included in augmentation of laboratory colonies or released.

In 1980, following the quarantine and mass‐rearing periods in USA: Florida, 50 *N. bruchi *adults were released from USA: Florida in Wallisville Reservoir, Texas, USA (Confrancesco, 1981). *N. eichhorniae *were found in this same reservoir as a consequence of westward migration from a biological control site in Louisiana (Confrancesco, 1981). In 1981, 500 adults of *N. eichhorniae *were imported from Louisiana populations and released in Wallisville, Texas (Shipper File No. CEVMS AFC 1981 1, USDA ARS, 2018). A total of 7,500 *N. eichhorniae *and 2,823 *N. bruchi *from the populations in Wallisville Texas were then released across four locations in the Sacramento–San Joaquin River Delta in California (Akers et al., 2017; Stewart et al., 1988). All other importation data pertinent to this study are summarized in Figure 1 and further detailed in the Supporting Information Appendix S1.

### 2.2 Specimen collections and DNA extraction

We collected *N. bruchi *and *N. eichhorniae *from: (a) the native range in the Uruguay River, Soriano, Uruguay, (b) the Sacramento–San Joaquin River Delta, California, USA; (c) Gainesville, Florida, USA; (d) Wallisville, Texas, USA; (e) Jilliby, Australia; (f) the Western Cape, Eastern Cape and KwaZulu‐Natal regions of South Africa, (g) Lake Victoria, Port Bell, Uganda; (h) Heping, Shantou, China; and (i) the Sungei Buloh Wetlands, Singapore (Table 1, Figure 1). We were unable to collect weevils from Argentina for this study due to the current limitations on biological exports in that country. Additional regions in the native (Argentina) and non‐native (Benin, Zimbabwe, and Thailand) range were not surveyed, but have documented importation pathways to several of the above populations and are thus included in Figure 1.

Weevils were preserved in 95% ethanol immediately after collection in the field. Prior to DNA extraction, we made photographic vouchers and catalogued lateral, ventral, and dorsal photographs for all weevils and uploaded onto a public Google Drive folder (Hopper, 2018). We extracted DNA using a modified Chelex extraction method from (Hopper et al., 2017). Purified DNA extractions were stored at −20°C until amplification with PCR. A total of 438 weevils were processed for DNA extraction (Table 1).

**Table 1:** Sampling information for *Neochetina bruchi *(*N. bruchi*) and *Neochetina eichhorniae *(*N. eichh*)

Location	Site ID	Study site	Collection	Latitude	Longitude	*N_N. bruchi_*	*N_N. eichh_*
Australia	AU	Jilliby	Jun‐2016	−33.224799	151.377455	21	20
China	CH	Heping, Shantou, Guangdong	Jun‐2017	23.251531	116.480457	–	23
Singapore	SI	Sungei Buloh Wetlands	Jul‐2017	1.440000	103.734633	–	33
SA: George	SAG	George, Western Cape	May‐2016	−34.031966	22.450090	–	22
SA: Wolseley	SAW	Wolseley, Western Cape	May‐2016	−33.424800	19.183700	6	23
SA: Enseleni	SAE	Enseleni Reserve, KwaZulu‐Natal	Aug‐2016	−28.688611	32.010556	18	–
SA: Kubusi River	SAK	Kubusi River, Eastern Cape	Sep‐2016	−32.564722	27.488889	–	22
Uganda	UG	Port Bell, Lake Victoria	Feb‐2017	0.289963	32.654657	26	26
Uruguay (Origin)	UR	Uruguay River, Soriano Department	Nov‐2016	−33.641392	−58.419423	29	31
USA: California	CA	San Joaquin River at 132	Oct–Dec 2015	37.641917	−121.228889	25	24
		Riverdale Park		37.612583	−121.038500		
USA: Florida	FL	Gainesville (USDA)	May‐2016	29.634337	−82.371453	21	21
USA: Texas	TX	Wallisville	May‐2017	29.837687	−94.761197	25	22

Note

“*N*” is the number of weevils used in microsatellite analyses.

### 2.3 Microsatellite marker development, genotyping, and analysis

Potential microsatellite loci for *N. bruchi *and *N. eichhorniae* were identified using a Perl script, PAL_FINDER_v0.02.03 (Castoe et al., 2012), and Primer3 (Rozen & Skaletsky, 2000) to analyze 150‐bp paired‐end Illumina sequences from extracted DNA enriched for microsatellite loci at the Savannah River Ecology Laboratory (University of Georgia, USA). From this, primers were designed for 48 loci, using only those with tri‐ and tetranucleotides and those with at least six repeats. For each species, the final loci for analysis were tested on DNA extractions from 24 adult weevils ranging across several collection sites. A set of 10 and 11 microsatellite loci for *N. bruchi *and *N. eichhorniae, *respectively, met the criteria of selection, that is, pure repeat, polymorphism, and amplification by PCR. Following amplification by PCR, eight and 10 loci, respectively (Table S1), were kept for the statistical analysis due to the high occurrence of null alleles in two loci for *N. bruchi *and one locus in *N. eichhorniae*.

After the initial screening, primers were combined in three multiplex reactions per individual for each species. For each 96‐well plate, we included a negative control (using water instead of DNA template) and an internal control of aliquoted DNA from an individual weevil that was used on every plate for the respective species. PCR multiplex reactions were run separately for the two species to avoid cross‐contamination. Pig‐tails (Table S1: GT, GTT, or GTTT) were added to the 5′ end of each reverse primer, and one of four different universal tails (Blacket, Robin, Good, Lee, & Miller, 2012) was added to the 5′ end of each forward primer (Table S1). The system of universal tailed primers was used to introduce a fluorescent dye during the PCR according to Blacket et al., (2012) and Culley et al. (2013). Initial singleplex and subsequent multiplex PCRs were in a final volume of 10 μl containing 50–70 ng of DNA, 5 μl of Qiagen Multiplex PCR Master Mix, 0.2 μM of reverse primer, 0.05 μM of forward primer, and 0.2 μM of the corresponding fluorescent primer using fluorescence‐labeled oligos (Life Technologies) (see Culley et al., 2013), and the addition of 2 μl of Qiagen Multiplex Q‐solution for several of the multiplex reactions (Table S1). PCR was performed at the following conditions: 95°C for 15 min; 35 cycles of 94°C for 30 s, the optimum annealing temperature (Ta) of each primer (Table S1) for 1.5 min, 72°C for 1 min, and a final extension of 30 min at 60°C.

Following successful amplification, 0.5 μl of the amplified product was added to 11 μl of solution containing 10.5 μl Hi‐Di formamide and 0.5 μl Liz size standard. Fragment lengths were measured in comparison with the GeneScan™ LIZ^®^ 600 Size Standard v. 2.0 (Life technologies) and genotyped on an Applied Biosystems 3730XL DNA Analyzer (Life Technologies) at the DNA Sequencing Facility at the University of California Berkeley. Fragment lengths were manually scored and binned using the Microsatellite Plug‐in for Geneious Pro v. 5.6.2 (Drummond et al., 2012). We re‐ran multiplex reactions and subsequently re‐genotyped samples if clear peaks were not obtained in the first run.

Genotype scores were checked with the program MICRO‐CHECKER v. 2.2.3 to identify possible null alleles and genotyping errors due to stuttering and large allele dropout (Van Oosterhout, Hutchinson, Wills, & Shipley, 2004).We re‐examined the relevant raw genotype data and either corrected the peak calls, or removed individuals that had poor quality peaks based on the recommendations of MICRO‐CHECKER. Genotype scores from the two weevil species were divided into two datasets for each species as the microsatellite markers did not overlap for weevils with diagnostic morphological characteristics for *N. bruchi *and *N. eichhorniae*. The dataset for *N. bruchi *consisted of genotype scores for 171 weevils from eight independent collection sites among five countries. The second dataset for *N. eichhorniae *consisted of genotype scores for 267 weevils from 11 independent collection sites among seven countries (Table 2). Final genotype scores for each individual, species, and collection site are in the Supporting Information Appendix S4.

**Table 2:** Genetic variability of *Neochetina bruchi *and *N. eichhorniae* at eight and 10 microsatellite loci, respectively, across collection localities from around the world

Sp	Population	*A*_R_	*A*_p_	*H*_E_	*H*_O_	*F*_IS_	*P*_HWE_	*g*_2_	*P*_g2_
*N. bruchi*	Australia	2.43	0	0.39	0.35	0.10	0.16	0.06	0.19
	SA: Wolseley	2.88	0	0.50	0.52	−0.04	0.38	0.01	0.47
	SA: Enseleni	3.13	3	0.54	0.42	**0.21**	**0.01**	0.02	0.35
	Uganda	2.53	1	0.42	0.40	0.05	**0.05**	0.01	0.48
	Uruguay	3.11	8	0.42	0.33	**0.22**	**0.00**	−0.05	0.83
	USA: California	2.76	1	0.45	0.38	**0.15**	0.09	**0.11**	**0.04**
	USA: Florida	2.63	0	0.42	0.36	0.14	0.49	0.10	0.10
	USA: Texas	2.56	0	0.42	0.39	0.07	**0.02**	−0.01	0.60
*N. eichhorniae*	Australia	3.42	0	0.48	0.42	0.12	0.05	−0.03	0.78
	China	2.74	0	0.45	0.46	−0.02	0.47	0.02	0.29
	Singapore	3.47	1	0.49	0.43	0.11	**0.00**	0.01	0.44
	SA: George	2.49	1	0.34	0.32	0.07	0.86	**0.11**	**0.05**
	SA: Wolseley	4.17	0	0.56	0.44	**0.22**	**0.00**	**0.10**	**0.02**
	SA: Kubusi River	4.02	0	0.55	0.5	0.08	0.08	−0.02	0.84
	Uganda	3.93	1	0.53	0.5	0.07	0.05	0.01	0.34
	Uruguay	5.16	10	0.56	0.5	0.10	**0.00**	0.04	0.09
	USA: California	4.00	4	0.53	0.54	−0.02	0.13	**0.11**	**0.05**
	USA: Florida	4.43	2	0.57	0.48	**0.15**	**0.00**	0.00	0.48
	USA: Texas	4.26	0	0.56	0.49	0.13	0.84	−0.01	0.67

Note

*A*_R_: allelic richness accounting for sample size; *A*_P_: private alleles unique to that location; *H*_E_: expected heterozygosity; *H*_O_: observed heterozygosity; *F*_IS_: fixation index; P_HWE_: *p* value from exact tests on the deviation from the Hardy–Weinberg equilibrium (HWE); g_2_, a parameter to test for inbreeding that measures the correlation of heterozygosity across pairs of loci; and Pg_2_, significant inbreeding based on g_2_. Bold values are significant or marginally significant.

We used the program GenAlex (Peakall & Smouse, 2006, 2012) and the R packages, “poppr” v. 2.5.0 (Kamvar, Brooks, & Grünwald, 2015; Kamvar, Tabima, & Grünwald, 2014) and “adegenet” (Jombart, 2008) to convert genotyping results into formats suitable for analysis in R (R Core Team, 2017). We calculated the null allele frequency (Brookfield, 1996) from the final datasets in the R package “popgenreport” (Adamack, Gruber, & Dray, 2014). As some statistical tests assume linkage equilibrium (LE) and Hardy–Weinberg equilibrium (HWE), we assessed deviations from LE with “poppr” and deviations from HWE across all sites for each locus (exact test) with the package “pegas” (Paradis, 2010). We constructed genotype accumulation curves with the R packages “poppr” and “vegan” (Oksanen et al., 2017) to test whether sufficient sampling had been performed for each species and collection site.

#### 2.3.1 Hybrid identification

To evaluate whether co‐introduction of these two related weevils species resulted in hybridization, we first identified individuals for each species that had ambiguous markings on the elytra that contrasted the typical morphological characteristics for that species (Figure S3). Then, we tested both sets of species‐specific microsatellite markers on 12 weevils with ambiguous morphological characteristics, as well as on weevils that had the typical species‐specific morphological characteristics for comparison. Hybridization is inferred from at least two of the species‐specific markers from each species amplifying in the same individual (see Weigel, Peterson, & Spruell, 2003).

#### 2.3.2 Effects of introduction processes on genetic variation and population structure

##### (2a–c) Genetic variation

As bottlenecks in population size can reduce genetic heterozygosity through processes of genetic drift and inbreeding, we estimated the average observed (*H*_o_) and expected (*H*_e_) heterozygosity, deviations from HWE (exact test), and the average “inbreeding coefficient” (*F*_IS_) for each collection site across all loci with the R package “diveRsity” (Keenan et al., 2013). Here, we use *F*_IS_ to estimate increases in homozygosity due to genetic drift caused by a larger population being separated into sub‐populations, rather than due to consanguineous mating (Crow, 2010). Thus, we used “g_2_” to test for inbreeding within populations of each weevil species by using 1,000 permutations in the R package “InbreedR”(Stoffel et al., 2016). In populations with inbreeding, g_2_ is significantly greater than zero, indicating correlated heterozygosity among pairs of loci (David, Pujol, Viard, Castella, & Goudet, 2007; Stoffel et al., 2016). We compared total and average allelic richness (accounting for sample size) and the number of private alleles among collection sites (R package “popgenreport”; Adamack et al., 2014).

To compare genetic diversity among the introduced and native populations, we tested for the effects of population (collection site) on genetic diversity by fitting linear mixed models (LMM) with the lmer function in the lme4 package (Bates, Maechler, Bolker, & Walker, 2015). Implementing an LMM accounts for the variability of the microsatellite loci by modeling locus as a random effect, and collection site as a fixed effect with allelic richness or expected heterozygosity as the response variables in separate models. Separate models were additionally used for each of the *Neochetina *spp. Stepwise model simplification (Crawley, 2013) was performed using likelihood ratio tests. Differences across collection sites were compared, based on 95% CI, using Tukey's posthoc test in the “multcomp” package (Hothorn, Bretz, & Westfall, 2008).

To determine whether the number of individuals released (propagule size) affects genetic diversity, we examined the number of weevils imported to each Florida, Texas, and California in the USA (described in the importation history) and the ratio of allelic richness retained from the native range in those states. To examine the influence of the number of introduction steps on the genetic diversity in populations of these two biological control agents, we combined the documented importation history (described previously and presented in Figure 1) and the genetic diversity data (Table 2). We counted the number of introduction steps based on the number of times the weevils were imported and exported since the initial export from the native range. We used linear models (LM) with the number of introduction steps from the native range for each population as a fixed effect with allelic richness or expected heterozygosity as the response variables in separate models. Separate models were used for each of the *Neochetina *spp.

##### (2d) Population genetic structure

We examined the population genetic structure of *N. bruchi *and *N. eichhorniae* to determine whether populations in the different locations have diverged from those in the native and introduced ranges since the initial introductions in the 1970s. Additionally, we explored whether the importation pathways impacted the population genetic structure of these two weevils. To initially examine whether population divergence has occurred, we conducted pairwise *F*_ST_ and Jost's *D* analyses with the R package “popgenreport” (Adamack et al., 2014). Although *F*_ST _is one of the most utilized metrics in population genetic studies, it can be biased downward for loci with multiple alleles (Meirmans & Hedrick, 2011). Thus, we additionally present Jost's *D* (Jost, 2008), which measures the fraction of allelic variation among populations, but can be biased upwards (Meirmans & Hedrick, 2011).

We used three additional population structure analyses to infer population structure by determining the number of genetic clusters (populations) and to assign individuals to their appropriate genetic cluster. We validate the results from these analyses by using information gained from the documented importation history. We used: (a) a discriminant analysis of principal components (DAPC) (Jombart, Devillard, & Balloux, 2010) with the package “adegenet” in R, (b) an iterative reassignment of individuals with the FLOCK software (Duchesne & Turgeon, 2012), and (c) a Bayesian approximation with the STRUCTURE software STRUCTURE (Pritchard, Stephens, & Donnelly, 2000). The FLOCK software and DAPC do not assume HWE or LE, in contrast to the program, STRUCTURE (Pritchard et al., 2000). As some of the microsatellite loci and populations in this study significantly deviated from HWE and LE (Supporting Information Appendix S1), we present the methods for analysis in the STRUCTURE program and the results from STRUCTURE in the Supporting Information Appendix S5.

The FLOCK software (Duchesne & Turgeon, 2012) first randomly divides all of the genotypes into *K* genetic groups (ignoring the sample memberships) and then reassigns the genotypes at each iteration to the group with the highest probability of belonging, using the multilocus method of maximum likelihood described by Paetkau, Calvert, Stirling, and Strobeck (1995). FLOCK was run both to provide an estimate of the number of populations and to determine which of a potential set of genetic sources is most likely the true source of each introduced population of both species. We used the plateau record to determine the estimate of *K* as described in Duchesne and Turgeon (2012). Default parameter values were used (20 reallocations per run, 50 runs) for each *k*.

To identify the most likely sources of an introduced population, we followed a systematic search procedure. In summary, we ran FLOCK with the novel sample and all plausible source samples while *k* was set at 2. Based on the resulting allocation tables from this run, all of the possible sources that were not mainly allocated to the same cluster as the novel sample were discarded. This same procedure was applied iteratively until only one potential source sample remains. When selecting the initial set of candidate sources for the allocation tables, we discarded the samples that could not be realistically considered potential sources. Those decisions were based mainly a priori on strong historical evidence. The searching procedure is described more formally and in greater detail in Supporting Information Appendix S2. When the searching procedure did not produce an unambiguous output, it was complemented by visualization with a DAPC run with the same samples (Supporting Information Appendices S2 and S3). We compared the DAPC results and FLOCK runs to the importation history (Figure 1).

## 3 RESULTS

### 3.1 Hybridization

We confirmed hybridization between *N. bruchi *and *N. eichhorniae* by analyzing the species‐specific markers on 12 individuals that had noticeable hybrid‐like markings on their elytra (Figure S3). One individual from California gave 100% amplification of microsatellite markers designed for *N. bruchi *and 80% of markers designed for *N. eichhorniae*, suggesting it may have been a first‐generation hybrid (F_1_). A second individual from Uruguay yielded 63% amplification of the markers designed for *N. bruchi *and 100% of markers designed for *N. eichhorniae*. Amplification of loci from both species in other individuals from populations in Texas and Uganda suggested possible later generation hybrid backcrosses (F_2_ or later), with 100% amplification of loci for one species and 30%–40% amplification of markers designed for the other species. As discussed in the methods, none of the species‐specific microsatellite markers developed for *N. bruchi* cross‐amplified on individuals with species‐specific morphological characteristics of *N. eichhorniae* and vice versa. Based on the morphological characteristics, we also noticed potential hybrids from the SA: George population, but these individuals did not amplify well for either set of markers likely due to poor DNA extractions. We could not analyze the prevalence of hybrids due to the sampling bias from collectors that selected individuals for each species based on distinct markings that separate the species.

### 3.2 (2a–c) Consequences of introduction processes on genetic variation

Allelic richness and expected heterozygosity did not differ significantly among populations of *N. bruchi* (Table 3, allelic richness, *χ* ^2^ = 11.03, *df* = 7, *p* = 0.14; H_E_ *χ* ^2^ = 6.89, *df* = 7, *p* = 0.44 respectively). In contrast, there was a significant effect of collection site on the allelic richness of *N. eichhorniae *(LMM, *χ* ^2^ = 47.00, *df* = 10, *p* < 0.001) and expected heterozygosity (*χ* ^2^ = 21.51, *df* = 10, *p* = 0.02). The lowest allelic richness and heterozygosity were found in George in the Western Cape, South Africa (SA: George), with the highest allelic richness occurring in Uruguay, and the highest heterozygosity in USA: Florida (Table 3). Uruguay had significantly higher allelic richness than Australia, China, SA: George and Singapore; and populations from USA: Florida, Texas, and SA: Wolseley had significantly higher allelic richness compared to China and SA: George (post hoc Tukey, *p < *0.05). SA: George had significantly lower expected heterozygosity compared to USA: Florida, Texas and SA: Wolseley and Uruguay (post hoc Tukey, *p < *0.05).

**Table 3:** Pairwise *F*_ST _and Jost's *D* values based on eight microsatellite loci from eight *Neochetina bruchi *collection localities

	Australia	USA: California	USA: Florida	SA: Wolseley	SA: Enseleni	USA: Texas	Uganda	Uruguay
*F*_ST_								
Australia	0.00							
USA: California	0.03	0.00						
USA: Florida	0.02	0.04	0.00					
SA: Wolseley	0.04	0.04	0.04	0.00				
SA: Enseleni	0.07	0.05	0.08	0.04	0.00			
USA: Texas	0.10	0.05	0.09	0.10	0.08	0.00		
Uganda	0.01	0.03	0.03	0.04	0.07	0.09	0.00	
Uruguay	0.06	0.04	0.08	0.05	0.09	0.07	0.06	0.00
Jost's *D*								
Australia								
USA: California	0.03							
USA: Florida	0.02	0.04						
SA: Wolseley	0.05	0.07	0.06					
SA: Enseleni	0.11	0.08	0.13	0.08				
USA: Texas	0.14	0.07	0.12	0.24	0.15			
Uganda	0.00	0.04	0.02	0.06	0.12	0.13		
Uruguay	0.08	0.05	0.11	0.12	0.16	0.10	0.08	0.00

Note

Underlined values are significant (more than or equal to 0.2)

We found increased homozygosity due to genetic drift, inbreeding or both in several populations for both *Neochetina *spp. Evidence of increased homozygosity, based on *F*_IS_ > 0.2, was found in the SA: Enseleni and Uruguay populations of *N. bruchi *and in the SA: Wolseley population of *N. eichhorniae*. Additional potential evidence of genetic drift was found in the California population of *N. bruchi* with *F*_IS_ = 0.15 (Table 3). Similarly, in California, USA, we found indications of potential inbreeding in both populations of both *N. bruchi *and *N. eichhorniae *(*p* < 0.05, Table 3) based on the g_2_ parameter. Additional evidence of inbreeding from the g_2_ parameter was found in two populations of *N. eichhorniae *in the Western Cape of South Africa (SA: George, and SA: Wolseley) (*p* ≤ 0.05, Table 3).

Overall, we did not find a correlation between the number of individuals released (propagule size, Lockwood et al., 2013) and the present‐day genetic diversity. For example, although the number *N. eichhorniae *released in USA: Florida (those initially imported from Argentina) was 14% that of the number of *N. bruchi* released*, *the retention of allelic richness from Uruguay in populations in USA: Florida was very similar (86% and 85% for *N. eichhorniae *and *N. bruchi, *respectively, Table 2). Similarly, in Texas, both weevil species demonstrated similar retention of allelic richness compared to the native range even though the propagule size of *N. bruchi *was 10% of the propagule size of *N. eichhorniae*. In fact, in California, we found a greater retention of allelic richness from the native range in populations of *N. bruchi *compared to *N. eichhorniae, *even though the number of *N. bruchi* imported from Texas to California was 38% that of *N. eichhorniae*. Furthermore, no clear effects of propagule size were observed in relation to genetic heterozygosity.

In addition to the absence of an effect of propagule size, we did not find a significant correlation between the number of introduction steps from the native range and allelic richness for *N. bruchi *(LM, F_1,5_ = 0.13, *p* = 0.74) or for *N. eichhorniae *(LM, F_1,8_ = 0.53, *p* = 0.49) (Figure S4a). Similarly, we did not find a significant correlation between the number of introduction steps from the native range and expected heterozygosity for *N. bruchi *(LM, *F*_1,5_ = 0.04, *p* = 0.85) or for *N. eichhorniae *(LM, *F*_1,8_ = 0.73, *p* = 0.42) (Figure S4b). For example, although Uganda had the highest number of introduction steps, it did not have the lowest allelic richness or expected heterozygosity (Figure S4a,b).

### 3.3 (2d) Population genetic structure of *N. bruchi*

Pairwise *F*_ST_ estimates were generally low for *N. bruchi *(<0.2), and the highest *F*_ST_ values (≥0.09) occurred in pairwise comparisons of *N. bruchi *genotypes from each Australia, USA: Florida, SA: Wolseley and Uganda against *N. bruchi *genotypes from USA: Texas, and between genotypes from Enseleni compared to those from Uruguay (Table 3). Overall Jost's *D* pairwise values were higher than the *F*_ST_ estimates, but presented similar patterns, with the highest value of 0.24 between SA: Wolseley and USA: Texas, and pairwise estimates of ≥0.13 between genotypes from USA: Texas and each Australia, SA: Enseleni and Uganda (Table 3). Jost's *D* values of >0.10 were observed between pairwise comparisons between *N. bruchi *weevils from SA: Enseleni and weevils from each Australia, USA: Florida and Uganda, as well as pairwise estimates between *N. bruchi *weevils from Uruguay and weevils from each USA: Florida, SA: Wolseley, and SA: Enseleni sites (Table 3).

The results from the FLOCK runs are visualized in Figure 1c and detailed in the Supporting Information Appendix S2. The initial run with all of the *N. bruchi *collection sites resulted in a *K* = 2. However, the weevils from SA: Enseleni were split 50% between the two reference groups, and 28% of weevils from California assigned to one reference group and 72% of these weevils to the other. Following this, a separate FLOCK run without the SA: Enseleni population resulted in a *K* = 2, with 46% of weevils from California assigned to one reference group and 56% of the weevils assigned to the other reference group. These results indicate that genetic admixture occurs in these populations, likely as a consequence of the importation history. Due to the composite nature of these two populations, we removed both SA: Enseleni and California populations from the main FLOCK analyses. Analysis of the resulting allocation tables for *K* = 2 (without SA: Enseleni or California) demonstrated one genetic cluster with Texas and Uruguay, and the other cluster consisting of Australia, USA: Florida, SA: Wolseley and Uganda. In addition to these two main genetic clusters, further FLOCK runs demonstrated significant subpopulation structure. Three genetic sub‐clusters were found in the USA: Texas site and two genetic sub‐clusters for each of the Uruguay, California, USA: Florida, and SA: Enseleni sites.

Allocation tables from FLOCK runs determined UR1, a sub‐cluster in Uruguay as the genetic source of the two USA: Florida sub‐clusters, but also indicated significant differentiation occurred between USA: Florida and the native range (mean LLOD = 3.20, *p* < 0.05). Genetic sources to the three USA: Texas sub‐clusters were identified as USA: Florida, Uruguay (subpopulation UR2), or both, but with significant differentiations occurring between all three sub‐clusters from USA: Texas compared to their allocated genetic source (*p* < 0.001). Of the USA sub‐clusters, we determined one of the genetic sub‐clusters in USA: California (CA1) as genetically sourced from USA: Florida and the other sub‐cluster in USA: California (CA2) as sourced from USA: Florida and two sub‐clusters in USA: Texas (TX1 and TX3). Contrary to the FLOCK allocation table, additional DAPC analysis (Supporting Information Appendix S2) indicated that Uruguay (UR1 and UR2) rather than TX3 contributed genetic sources to the genetic sub‐cluster CA2. All of the FLOCK allocations demonstrated significant differentiation of the two sub‐clusters in USA: California from these source populations (*p* < 0.001).

The population in SA: Wolseley was genetically sourced (allocated) from populations in USA: Florida and Australia, with no genetic differentiation between the weevil population in SA: Wolseley and the populations from Australia and Florida (mean LLOD = 1.97, *p* = 0.29). We determined that the genetic sub‐cluster (SA: Enseleni‐1) was mostly allocated to an untested source, with more similarity to Uruguay than to USA: Florida or Australia (mean LLOD = 2.42, *p* < 0.001), but clearly genetically distinct from the population in Uruguay (mean LLOD = 3.06, *p* < 0.001). In contrast, analyses clearly pointed to the population from FL2, a genetic sub‐cluster in USA: Florida, as the source for the sub‐population, SA: Enseleni‐2 (mean LLOD = 2.18, *p* = 0.82), compared to the other populations (mean LLOD = 2.01, *p* < 0.001).

**Figure 2:**
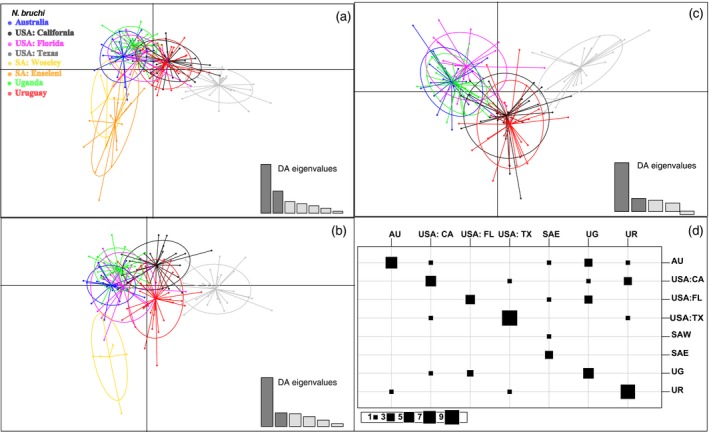
Discriminant analysis of principal components (DAPC) stepwise reduction of relationships based on eight microsatellite genotypes among eight collection localities of *Neochetina bruchi*. Individuals are color‐coded based on location. The first two principal components are shown for each of the three DAPC analyses: (a) all collection localities, (b) SA: Wolseley population is removed from analysis, (c) both SA: Wolseley and SA: Enseleni populations are removed from the analysis; and (d) a contingency table from the DAPC analysis utilizing all populations, with the columns representing the actual clusters of supplementary individuals and rows representing the inferred clusters based on the predictions of the DAPC analysis (65% accuracy). Abbreviations are described in Table 1

In support of FLOCK analyses, DAPC also found two main genetic clusters and further population substructure. DAPC indicated a clear separation of *N. bruchi* collected from Texas compared to the weevils collected from Australia, SA: Wolseley, SA: Enseleni, and Uganda (Figure 2). In addition to supporting the majority of the results from *F*_ST_ and Jost's *D* analyses and FLOCK runs (Figure 2), the DAPC clarified population structure when FLOCK analyses were unclear (Supporting Information Appendix S2).

### 3.4 (2d) Population structure of N. eichhorniae

The highest *F*_ST _pairwise values ranged from 0.19 to 0.24 for Singapore and China compared to the population from SA: George, respectively (Table 4). Overall Jost's *D* pairwise values were higher than *F*_ST_ estimates, but presented similar patterns, with high values of >0.2 for pairwise estimates of all of the sites compared to Singapore, and values of >0.2 for China in each pairwise comparison to USA: California, USA: Florida, Singapore, two sites in South Africa (SA: George and SA: Kubusi River) and Uruguay. Additionally, Jost's *D* indicated distinct structure (>0.2) between SA: George and Texas and SA: George and Uganda (Table 4).

**Table 4:** Pairwise *F*_ST _and Jost's *D* values based on 10 microsatellite loci from 11 *Neochetina eichhorniae *collection localities

	Australia	China	USA: California	USA: Florida	Singapore	SA: George	SA: Wolseley	SA: Kubusi River	USA: Texas	Uganda	Uruguay
*F*_ST_											
Australia	0.00										
China	0.11	0.00									
USA: California	0.03	0.12	0.00								
USA: Florida	0.03	0.11	0.03	0.00							
Singapore	0.09	0.10	0.11	0.09	0.00						
SA: George	0.11	0.24	0.07	0.08	0.19	0.00					
SA: Wolseley	0.04	0.08	0.03	0.03	0.10	0.11	0.00				
SA: Kubusi River	0.04	0.11	0.03	0.03	0.11	0.10	0.03	0.00			
USA: Texas	0.03	0.08	0.03	0.03	0.09	0.12	0.02	0.03	0.00		
Uganda	0.04	0.10	0.04	0.04	0.10	0.12	0.02	0.04	0.04	0.00	
Uruguay	0.03	0.13	0.04	0.02	0.10	0.09	0.05	0.04	0.05	0.07	0.00
Jost's *D*											
Australia	0.00										
China	0.20	0.00									
USA: California	0.03	0.25	0.00								
USA: Florida	0.04	0.24	0.04	0.00							
Singapore	0.17	0.20	0.25	0.20	0.00						
SA: George	0.16	0.41	0.11	0.14	0.36	0.00					
SA: Wolseley	0.06	0.16	0.05	0.05	0.23	0.19	0.00				
SA: Kubusi River	0.05	0.21	0.04	0.05	0.25	0.17	0.04	0.00			
USA: Texas	0.05	0.16	0.04	0.07	0.21	0.22	0.02	0.04	0.00		
Uganda	0.06	0.19	0.06	0.08	0.23	0.21	0.04	0.08	0.06	0.00	
Uruguay	0.05	0.29	0.08	0.04	0.25	0.17	0.13	0.08	0.11	0.15	0.000

Note

Underlined values are significant (more than or equal to 0.2)

The results from the FLOCK runs are visualized in Figure 1d and detailed in the Supporting Information Appendix S3. Plateau analyses from the FLOCK runs indicated a total of four to six distinct populations (*K* = 4 to *K* = 6). The initial analysis separated the genetic cluster consisting of China and Singapore from the populations including: Australia, all three sites in the USA (Florida, California, Texas), all three sites in South Africa (SA: George, SA: Wolseley, SA: Kubusi River), Uganda, and Uruguay. Separate FLOCK runs with just the China and Singapore sites determined that these two populations were genetically distinct from one another, each as a distinct genetic cluster (*K* = 2, plateau length of 50, mean LLOD = 5.23, *p* < 0.001). After removing China and Singapore populations from the main analysis, further FLOCK runs resulted in a plateau analysis that indicated the potential of two additional genetic clusters (plateau analysis was undecided between *K* = 1 and *K* = 2). The majority of *N. eichhorniae* from California, Florida, SA: Wolseley, SA: Kubusi River, Texas, and Uganda formed one genetic cluster, and the majority *N. eichhorniae* from Uruguay and all of the weevils from SA: George formed the second genetic cluster, with weevils from Australia split equally between these two genetic clusters. We found additional subpopulation structure in FLOCK for populations of *N. eichhorniae*, indicating two genetic sub‐clusters within SA: George, SA: Wolseley, Singapore, USA: Texas, and Uruguay; two to three genetic sub‐clusters within the USA sites: California and Florida; and three sub‐clusters within the China, SA: Kubusi and Uganda sites (Supporting Information Appendix S3).

Results from FLOCK allocation tables clarified the genetic sources of several of the examined populations of *N. eichhorniae*. In the USA, one of the California sub‐clusters (CA1) was determined to be genetically sourced from and identical to the population from USA: Florida and a USA: Texas subpopulation (TX2) (mean LLOD = 2.66, *p* = 0.52), and this was further supported by DAPC (Supporting Information Appendix S3). The most likely genetic source to the second USA: California sub‐cluster (CA2) was from a second USA: Texas sub‐cluster (TX1) rather than from USA: Florida or Uruguay, but CA2 still significantly differed from the genetic sub‐cluster TX1 (mean LLOD = 2.41, *p* < 0.001). The two USA: Texas sub‐clusters (TX1 and TX2) were each determined to be genetically sourced from USA: Florida (mean LLOD = 2.99, *p* = 0.72; mean LLOD = 2.80, *p* = 0.71, respectively). As DAPC analyses indicated the two USA: Florida sub‐clusters were not very different, we used the whole USA: Florida population in allocation tables in FLOCK to determine the genetic source. The population in USA: Florida was determined to have genetic sourcing from UR2, a Uruguayan sub‐cluster rather than from UR1 (mean LLOD = 3.07, *p* < 0.01).

The population in Uganda was most likely genetically sourced from Australia, but still differed in genetic composition (mean LLOD = 2.52, *p* = 0.02). The population in Australia was most likely genetically sourced from USA: Florida, but still significantly differed in genetic composition (mean LLOD = 2.49, *p* = 0.01). DAPC supported these FLOCK analyses and demonstrated that genetic sources from the Uruguayan subpopulation UR2 went to USA: Florida and to Australia. In South Africa, *N. eichhorniae *from SA: George had the highest genetic similarity to the weevils from Australia compared to the other potential source populations, but still differed significantly indicating additional genetic contribution from an untested source (SA: George compared to AU; mean LLOD = 3.28, *p* < 0.001). Weevils from SA: Wolseley were most likely genetically sourced from the population from USA: Florida, but the genetic structure between these two populations still differed significantly (mean LLOD = 2.56, *p* < 0.001). The weevil population from SA: Kubusi River was determined to be genetically sourced and identical to the populations from both USA: Florida and Australia (mean LLOD = 2.82, *p* = 0.12). Populations in China and Singapore were not genetically sourced from any of the tested populations (mean LLOD = 3.63, *p* < 0.001). Furthermore, the weevils in these populations significantly differed from each other, thus indicating different importation histories and pathways to these populations (mean LLOD = 5.23, *p* < 0.001).

**Figure 3:**
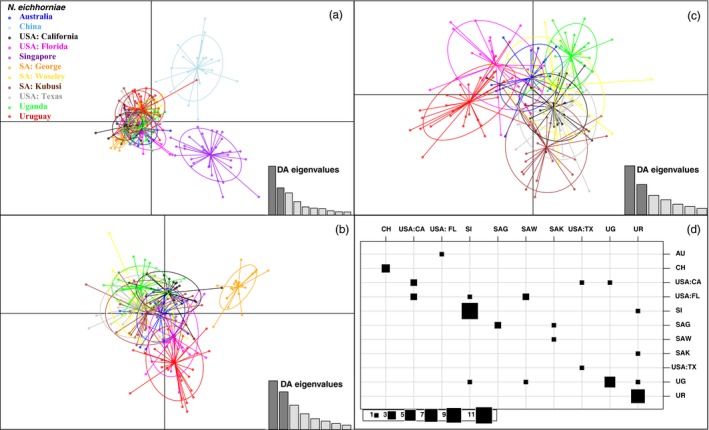
Discriminant analysis of principal components (DAPC) stepwise reduction of relationships based on 10 microsatellite genotypes among 11 collection localities of *Neochetina eichhorniae*. Individuals are color‐coded based location. The first two principal components are shown, with three DAPC analyses: (a) all collection localities, (b) China and Singapore are removed from analysis, (c) China, Singapore, and SA: George population are removed from analysis; and (d) a contingency table from the DAPC analysis utilizing all populations, with the columns representing the actual clusters of supplementary individuals and rows representing the inferred clusters based on the predictions of the DAPC analysis (68% accuracy). Abbreviations are described in Table 1

The DAPC for *N. eichhorniae* (Figure 3) indicated six main genetic clusters, supporting the FLOCK runs that indicated 4–6 populations. Initial DAPC depicted a clear separation and genetic clustering between weevils collected from China and weevils collected from Singapore, as well as a separation of these two clusters from the weevils collected from the USA, the three sites in South Africa, Australia, and Uruguay. When China and Singapore clusters were removed from the DAPC, a separation between weevils from SA: George and the other sites was also observed. After removing SA: George from the DAPC analysis, differences between Uruguay and Uganda were observed (Figure 3). In addition to supporting the majority of the results from *F*_ST_ and Jost's *D* analyses, and FLOCK runs, the DAPC clarified population structure when FLOCK analyses were unclear (Supporting Information Appendix S3).

## 4 DISCUSSION

Here, we examined the genetic diversity in and among populations of two widespread biological control agents of water hyacinth, the weevils: *N. bruchi *and *N. eichhorniae*.

Among the most striking results was the confirmation of hybridization between *N. bruchi *and *N. eichhorniae*. We found interspecific hybrids in California, Uruguay, and Uganda, ranging from potential first‐generation hybrids (F_1_) to F_2_ and later generations. Based on the morphological characteristics, we also noticed potential hybrids from South Africa, but these individuals did not amplify well for either set of markers likely due to poor DNA extractions. Interspecific hybrids are likely present in all regions based on the fact that we found a hybrid from a population in Uruguay in the native range, and they may have been introduced from the original collections and releases. However, we could not accurately assess the percent of hybrids per site due to the strong likelihood of sampling bias against hybrids during the collections. Future studies should conduct in‐depth surveys to examine the prevalence of hybrids in the native and introduced regions and perform hybrid crosses. As these two weevils are used across the globe for the biological control of water hyacinth, it is important to investigate the effect of hybridization on the performance and growth of these weevils. Interspecific hybrid crosses can result in hybrid vigor or hybrid breakdown (Arcella et al., 2014; Bean et al., 2013) as well as affect the host‐specificity of a biological control agent (Bitume et al., 2017; Mathenge et al., 2010). If fitness of hybrids is low, it may be useful to determine the conditions under which hybrids form and try to minimize hybridization in regions where biological control programs are critical for the control of water hyacinth.

In addition to the occurrence of hybridization, we found evidence of genetic drift and inbreeding in several populations. From the importation history, there is documented evidence that these weevils went through demographic bottlenecks during the importation and release phases of the biological control programs. Subsequent drift or inbreeding following demographic bottlenecks can lead to increased homozygosity (Crow, 2010). We found evidence of genetic drift in the SA: Enseleni and Uruguay populations of *N. bruchi *and in the SA: Wolseley population of *N. eichhorniae*. The occurrence of genetic drift in the native range was unexpected as allelic richness was the highest in Uruguay and genetic drift typically occurs in populations that have undergone a demographic bottleneck (Bock et al., 2015; Nei et al., 1975). Alternatively, these results may have been artifacts of marker scoring (see David et al., 2007), the sampling or the markers used in this study (Selkoe & Toonen, 2006). Additional potential evidence of genetic drift or inbreeding was found in the California population of *N. bruchi* with *F*_IS_ = 0.15 (Table 3). We also found indications of potential inbreeding in both populations of *N. bruchi *and *N. eichhorniae *in California and in two populations of *N. eichhorniae *in the Western Cape of South Africa (SA: George, and SA: Wolseley) (Table 3). Although inbreeding can have detrimental consequences, it has also been known to promote local adaptation (Verhoeven et al., 2011).

Integrating the estimates of genetic diversity with the importation history for these biological control agents also permitted us to examine the consequences of propagule size and introduction processes on the genetic diversity of introduced populations. We did not find any evidence that initial propagule size or the number of introduction steps affected current day genetic diversity in populations of *N. bruchi *or *N. eichhorniae*. However, initial propagule sizes in this study system may have been higher than a specific threshold required for an effect to have taken place. Our initial hypothesis that populations with more introduction steps away from the native range would harbor lower genetic diversity than those populations with fewer steps was not supported. For example, we found intermediate levels of genetic diversity in Uganda for both species even though the populations in Uganda had the highest number of steps away from the native range. Overall, *N. bruchi *had similar allelic richness and heterozygosity across the eight collection sites. Although not significantly higher, the populations of *N. bruchi *in the native range (Uruguay) and in SA: Enseleni harbored the most alleles. In contrast, there was significant variation in allelic richness and expected heterozygosity across populations of *N. eichhorniae, *with the highest allelic richness in the population in Uruguay. Populations of each *N. bruchi *and *N. eichhorniae* from Uruguay also exhibited the highest number of private alleles, with eight and 10 private alleles, respectively. The high allelic richness and many private alleles found in the population in Uruguay supports the general trends that introduced populations typically undergo a loss in genetic diversity (Dlugosch et al., 2015; Dlugosch & Parker, 2008), but see (Estoup et al., 2016; Goodnight, 1988; Kolbe et al., 2004; Taylor et al., 2011).

We were also able to investigate the potential effects of genetic admixture on genetic diversity as a result from multiple introductions that occurred in this study system. The FLOCK allocation tables generally reflected the movement of weevils documented in the importation records (Figure 1) and additionally clarified genetic sources where the importation history was unclear. In places such as South Africa, the importation history was unclear due to intervening importations from multiple locations and multiple introductions across the country. Interestingly, one of the populations of *N. bruchi *in South Africa, SA: Enseleni, had equivalent allelic richness to the population in the native range. Although the other population (SA: Wolseley) had lower allelic richness, the sample size for that population was only six individuals. FLOCK allocation tables helped demonstrate that SA: Enseleni was a composite population with two genetic sub‐clusters. One sub‐cluster was mostly allocated to the Australian genetic cluster, and the other cluster appeared somewhat related to the Australian and Ugandan genetic clusters. Based on the importation history, the latter sub‐cluster was likely derived from Zimbabwe, a population that we did not test. This finding supported the multiple introductions documented in the importation history, and the notion that genetic admixture can increase genetic diversity (Rius & Darling, 2014). Furthermore, genetic admixture may be able to rescue populations that had small initial propagule size or underwent demographic bottlenecks (Hufbauer et al., 2015) Additionally, two out of the three populations of *N. eichhorniae *in South Africa demonstrated high allelic richness (>4) and FLOCK allocation tables found that one of these populations (SA: Kubusi River) had two genetic sources (Australia and USA: Florida). Interestingly, FLOCK analyses demonstrated that only one population from USA: Florida contributed to the genetic composition of SA: Wolseley, which also demonstrated high allelic richness. This population also had indications of genetic drift and inbreeding, which supports the contrasting forces of genetic admixture and inbreeding, with the latter sometimes selected for when a population is adapted to the local area (Verhoeven et al., 2011).

In contrast, the populations of *N. eichhorniae *in the USA also had high allelic richness, even though FLOCK allocation tables indicate a single introduction from the native range. It appears that only one Uruguayan sub‐cluster contributed to the current day genetic composition of USA: Florida. This was particularly interesting since the importation history indicates two populations from South America were initially imported to USA: Florida. However, the lack of genetic contribution from the two populations in the native range is likely due to the fact that only 10 individuals from Campana Lagoon were imported (in comparison with 156 individuals from Santa Fe, Argentina), due to a low abundance of *N. eichhorniae *in the Campana Lagoon. Although we sampled populations from Uruguay, we sampled them from the Uruguay River, in‐between Argentina and Uruguay, and speculate that the sample is likely genetically similar to those weevils in Santa Fe, Argentina. Thus, rather than due to multiple introductions, the higher allelic richness in Florida, Texas, and California, USA, may have been due to the temporal proximity of these populations to the initial imported population from the native range (even though multiple steps, and thus serial bottlenecks, occurred).

In addition to clarifying the introduction pathways, our population genetic analyses demonstrated the presence of several distinct and broad genetic clusters for each *N. bruchi *and *N. eichhorniae*. In the case of *N. bruchi*, FLOCK and DAPC indicated two main genetic clusters and 11 sub‐clusters For *N. eichhorniae, *FLOCK and DAPC signified four to six main genetic clusters and 23 sub‐clusters. In comparison, the STRUCTURE program detected two to six distinct broad populations for each weevil species, but did not detect sub‐clustering within these populations (Supporting Information Appendix S5, Figures S2 and S3). This indicates that significant divergence occurred among and between several of the introduced populations and the native population since the initial introductions in the 1970s. This supports previous studies on invasive species and biological control agents that demonstrate the divergence of populations from the native range (Zepeda‐Paulo et al., 2015) but see Franks et al. (2010). Divergence of introduced populations from the native populations likely depends on the time since the initial introduction. For example, we sampled populations almost 50 years after the initial introductions, whereas Franks et al. (2010) sampled in the introduced range just 2 years after the initial releases.

One caveat that we acknowledge is that the genetic divergence between the introduced and native range may have been due to the fact we sampled from Uruguay rather than Argentina, where the actual initial source populations were exported from. However, based on the DAPC and FLOCK analyses, the populations from Uruguay for both species appear to be genetic sources for several of our populations. Thus, we feel confident that the genetic composition from weevils in Argentina compared to those in Uruguay is not very different.

In addition to the results demonstrating that genetic drift and inbreeding occurred in several populations, we speculate that divergence has also occurred due to local adaptation to some of the regions of introduction. Rapid local adaptation has been observed in invasive species (Sotka et al., 2018) as well as in biological control agents (Phillips et al., 2008). Many of the introduced regions that we tested in this study have colder climates than that occurring in South America. Recently, Reddy et al. (2018) tested the cold‐temperature tolerance and life‐history performance of *N. eichhorniae *under cool temperature conditions simulating the fall season in Sacramento–San Joaquin River Delta, California. Reddy et al. (2018) tested the same populations of *N. eichhorniae *used in the present study and found that weevils from the population in Australia had a higher fecundity under these cool temperature conditions compared to weevils from California and Uruguay, SA: Kubusi River. These results were surprising as the population in Australia had lower genetic diversity than the other populations, thus suggesting that populations can still adapt to local areas even with moderate levels of genetic diversity. Furthermore, the present study combined with that of Reddy et al. (2018) demonstrates that both genetic composition and life‐history performance may have diverged among these populations.

We support the recommendation that population genetic analyses be performed prior to the selection and release of biological control agents (see Rauth, Hinz, Gerber, & Hufbauer, 2011). The genetic diversity and genetic composition may have implications for the population growth of the biological control agents and their success in controlling the target weed or pest. Although these weevils have shown tremendous success in reducing water hyacinth in a number of countries (Julien et al., 2000), less than optimal levels of control has been found in regions with cooler temperatures, including some of the high altitude areas in South Africa (Hill & Olckers, 2001; May & Coetzee, 2013) and in the Sacramento–San Joaquin River Delta, in northern California (Hopper et al., 2017). The lower efficacy of biological control in these regions could be due to climatic mismatch and/or the inability to thrive and adapt to the local area based on the genetic diversity and composition as influenced by importation methods and the selected source populations.

## CONFLICT OF INTEREST

None declared.

## DATA ARCHIVING STATEMENT

1. Microsatellite allele calls used for this study are available in Supporting Information Appendix S4, accompanying this article.
2. Photographs of the weevils, *N. bruchi* and *N. eichhorniae*, are found in a public Google Drive folder: https://drive.google.com/drive/folders/0B6mDY592nSW9NWJLWDh5MVR3djA?usp=sharing.
