# Supplementary material for: Into the weeds: Matching importation history to genetic consequences and pathways in two widely used biological control agents
Source: Evol Appl. 2019 Jan 4;12(4):773–90. doi: 10.1111/eva.12755 (PMC6439500; doi:10.1111/eva.12755)
Supplement: Supplementary file 1 [file EVA-12-773-s001.docx]

*Additional details from the importation history*

In Jilliby Australia, it is estimated that 160-200 weevils comprised of a mixture of *N. bruchi* and *N. eichhorniae* were released in 1994 (personal communication, Matthew Purcell, USDA ARS Australian Biological Control Laboratory, via communication with Paul R Sullivan, Invasive Species Officer - Biocontrol NSW Department of Primary Industries, Vertebrate Pest and Weed Research Units, Tamworth Agricultural Institute, Calala NSW 2340).

In South Africa, releases of *N. eichhorniae* were initiated in 1974-1975 from a mixture of populations imported directly from Argentina with those from USA: Florida (Cilliers 1999). In 1985 these populations were additionally augmented with source populations from Australia (source from Argentina via USA: Florida) and released in 1985-86. Populations of *N. bruchi* in South Africa were initiated from imports of ‘small numbers’ from Florida and released in 1990, with additional importations from Zimbabwe (sourced from USA: Florida) in 1994 (Cilliers 1999; Winston *et al.* 2014). Up until 2008, all weevils were mass reared at the Protection Research Institute in Pretoria, South Africa and released around the country (Cilliers 1999). In 2008, the Sugar Research Institute in Kwazulu Natal took over mass rearing and further distributed the weevils around the country, using stocks from the Enseleni River (personal communication, Dr. Julie Coetzee, Rhodes University).

In Beijing, China, records denote two shipments amounting to a total of 100 *N. bruchi* and 285 *N. eichhorniae* sent in 1995 from Gainesville Florida, USA (Shipper File No. FBCL 1995 1 FBCL 1995 2,USDA ARS 2018). Following quarantine testing and mass rearing, a mixture of 1000 weevils (including *N. bruchi* individuals additionally imported from Argentina), were released in 1996 into the Wenzhou and Zhejiang provinces. These weevils were eventually redistributed around the country, including releases in 2001 into the Guangdong province (region of our China study site) (Julien *et al.* 2000).

**References**

Cilliers CJ (1999) Biological control of water hyacinth, Eichhornia crassipes (Pontederiaceae), in South Africa. *Agriculture, Ecosystems and Environment* **37**, 207-217.

Julien MH, Hill MP, Center TD, Jianqing D (2000) Biological and integrated control of water hyacinth, *Eichhornia crassipes* *Proceedings of the Second Meeting of the Global Working Group for the Biological and Integrated Control of Water Hyacinth*

USDA ARS (2018) Releases of Beneficial Organisms in the United States and Territories (ROBO) database. Biological Control Documentation Center.

Winston RL, Schwarzländer M, Hinz HL*, et al.* (2014) Biological Control of Weeds: A World Catalogue of Agents and Their Target Weeds (ed. Service UF), p. 838. Forest Health Technology Enterprise Team, Morgantown, West Virginia.

*Microsatellite data analyses*

We used a final set of eight and ten polymorphic microsatellite loci respectively for *N. bruchi* and *N. eichhorniae* (Table S1)*.* Genotype accumulation curves demonstrate near saturation for *N. bruchi* (Fig. S1) and complete saturation for *N. eichhorniae* (Fig. S2). The number of alleles per locus ranged from 2 to 10 in *N. bruchi* and from 4 to 16 in *N. eichhorniae*.

Significant deviation from Hardy Weinberg Equilibrium (HWE) was found in three of the eight *N. bruchi* collection sites, and four of the eleven *N. eichhorniae* collection sites when analyzed across all loci (exact test, P < 0.05, Table 2). When analyzed across all sites, six out of the eight loci for *N. bruchi* and seven of the ten loci for *N. eichhorniae* deviated from HWE (Exact test, P < 0.05, Table S2). The number of loci that significantly deviated from HWE varied based on collection site, with a maximum of two loci for *N. bruchi* deviating from HWE in Uruguay, and a maximum of four loci for *N. eichhorniae* deviating from HWE in Kluitjies Kraal, Western Cape of South Africa (SAB) (Exact test, P < 0.05). Loci not in HWE had excess/reduced polymorphism than if they had been in equilibrium.

Significant linkage disequilibrium was found for *N. bruchi* in one pairwise comparison of loci (Nb_27 and Nb_26) when analyzed across the eight collection sites (Table S3). A total of 16 out of 225 pairwise comparisons of loci significantly deviated from linkage equilibrium when analyzing each of the sites individually. For *N. eichhorniae*, significant linkage disequilibrium was found for five pairwise comparisons of loci when analyzed across the ten collection sites (Table S3), and for a total of 29 out of 495 pairwise comparisons of loci when analyzing each of the sites individually. Due to these deviations from equilibrium, we focused on the results from the FLOCK program and DAPC to analyze population structure and allocation rather than other programs that assume HWE and LE such as STRUCTURE.

The frequency of null alleles based on the Brookfield estimation method ranged from -0.01 for the Nb_13 locus to 0.17 for the Nb_40 and Nb_46 loci in *N. bruchi*, and ranged from 0.00 for the Ne_38 locus to 0.21 for the Ne_14 locus in *N. eichhorniae* (Table S4).

**Table S1** Primer sequences and PCR conditions for the microsatellite loci of *Neochetina bruchi* and *N. eichhorniae* in this study. Universal tails and pig-tails that were added to the forward and reverse sequences respectively are underlined. Primers are binned by their respective multiplex reactions, with the annealing temperature (**°**C) and a (Q) symbol indicating that the Qiagen multiplex Q solution was used in the PCR reaction mix.

| **Sp.** | **Locus** | **Primer Sequences 5'-3'** | **Dye** | **bp** | **Motif** | **(Q)/Ta°** |
| --- | --- | --- | --- | --- | --- | --- |
| ***N. bruchi*** | Nb_40 | F: GCCTCCCTCGCGCCAAGCCACTCGTGCTAGACTTC | FAM | 168-212 | AAT(10) | 60 |
|  |  | R: GTTTATCAGCAGCCTCAATAACCTC |  |  |  |  |
|  | Nb_46 | F: GCCTTGCCAGCCCGCTTGGTCAGGGTTGTGAGAAG | VIC | 217-244 | AGG(8) |  |
|  |  | R: GTTTCGAACACCGTGACAGTTAAAG |  |  |  |  |
|  | Nb_5 | F: GCCTTGCCAGCCCGCGTTTCCGGTTCAGGGTTGTG | VIC | 230-257 | AGG(9) | (Q) 64 |
|  |  | R: GTTTACCACCTACGCATAATCCC |  |  |  |  |
|  | Nb_27 | F: GCCTCCCTCGCGCCAGCAGACTTATCCGATCTCAAGG | FAM | 201-207 | AGG(8) |  |
|  |  | R: GTTTATTCTGTCAGGGTTGTGAGAC |  |  |  |  |
|  | Nb_43 | F: CAGGACCAGGCTACCGTGTTTCGAACCGCACAAGATCC | NED | 199-211 | AGG(8) |  |
|  |  | R: GTTTAGAACCTCCTCCTCTTGTCAG |  |  |  |  |
|  | Nb_8 | F: GCCTTGCCAGCCCGCTTGAGTTAGCTAGACTTCGCC | VIC | 263-290 | AAT(13) | (Q) 63 |
|  |  | R: GTTTCTCACAACCCTGACAAGAGG |  |  |  |  |
|  | Nb_13 | F: CGGAGAGCCGAGAGGTGTTTGTGAGGTCGCTGGTTAC | PET | 224-245 | AGG(10) |  |
|  |  | R: GTTTACAAGGAAATTCTGCCAGGG |  |  |  |  |
|  | Nb_26 | F: CAGGACCAGGCTACCGTGAGATGGGAAATGATGTTGCTTG | NED | 208-223 | AGG(8) |  |
|  |  | R: GTTTCTGCATGGAAATTCTGTCAGG |  |  |  |  |
|  |  |  |  |  |  |  |
| ***N. eichhorniae*** | Ne_14 | F: GCCTCCCTCGCGCCAAGACGTGTACCTCTTTCTGGAG | FAM | 188-209 | AGG (9) | 61.8 |
|  |  | R: GTTTAATTCTGCCAGGGTTGCAAC |  |  |  |  |
|  | Ne_26 | F: CAGGACCAGGCTACCGTGGTTGCTGAGGATGGGTGTTG | NED | 143-161 | AGG (8) |  |
|  |  | R: GTTTACTTGTTCCGTTCAGATTTGTG |  |  |  |  |
|  | NE_91 | F: GCCTTGCCAGCCCGCTTTTACATTGGCTTCTCATTGG | VIC | 284-352 | AAAT (28) |  |
|  |  | R: GTTTCAACTCAATCGATACGATATTCCC |  |  |  |  |
|  | NE_94 | F: CGGAGAGCCGAGAGGTGGCAAAAGTTATAGGGTAAAACACGC | PET | 182-214 | ATAG (24) |  |
|  |  | R: GTTTCAATAAAAGGGTCATCCTGG |  |  |  |  |
|  | Ne_13 | F: GCCTTGCCAGCCCGCACAAGGAAATTCTGCCAGGG | VIC | 276-288 | AGG (9) | (Q) 64 |
|  |  | R: GTTTGCTGTGTTTGTCTTTGAGAACC |  |  |  |  |
|  | Ne_17 | F: GCCTCCCTCGCGCCAGGTGCGGTCTTCCTATCTC | FAM | 194-222 | AGG (8) |  |
|  |  | R: GTTTAGAACCTCCTCCCAGAAATGG |  |  |  |  |
|  | Ne_42 | F: CAGGACCAGGCTACCGTGTGGGTTGTCTCAGGAAGCAG | NED | 235-253 | AGG (9) |  |
|  |  | R: GTTTGTCAGGGTTGCCACTAC |  |  |  |  |
|  | NE_56 | F: CGGAGAGCCGAGAGGTGAATTCTGTCAGGGTTGCCAC | PET | 189-204 | AGG (10) |  |
|  |  | R: GTTTGTAGAGATATTCGGCAGCG |  |  |  |  |
|  | Ne_8 | F: CAGGACCAGGCTACCGTGAATCCATGCTGTTCAGGAGG | NED | 183-222 | AAAT (6) | (Q) 64 |
|  |  | R: GTTTAGGGAGAGTGGGCAATTCTG |  |  |  |  |
|  | Ne_38 | F: GCCTTGCCAGCCCGCAATTCTGCCAGGGTTGCAAC | VIC | 246-255 | AGG (8) |  |
|  |  | R: GTTTCCGATAGCCCTTCTTTCAGTAG |  |  |  |  |

**Table S2** Probability of deviation of loci from Hardy-Weinberg Equilibrium (HWE) across all collection localities for *N. bruchi* and *N. eichhorniae*. P-values of deviation are based on exact tests using Monte Carlo permutations of alleles (1000 permutations).

| Sp. | Locus | P_HWE_ |
| --- | --- | --- |
| *N. bruchi* | Nb_40 | **0.00** |
|  | Nb_46 | **0.00** |
|  | Nb_8 | **0.00** |
|  | Nb_5 | 0.09 |
|  | Nb_27 | **0.01** |
|  | Nb_26 | **0.01** |
|  | Nb_13 | 0.32 |
| *N. eichhorniae* | Nb_43 | **0.03** |
|  | Ne_14 | 0.05 |
|  | Ne_91 | **0.00** |
|  | Ne_26 | **0.00** |
|  | Ne_94 | **0.00** |
|  | Ne_17 | **0.00** |
|  | Ne_13 | **0.00** |
|  | Ne_42 | **0.00** |
|  | Ne_56 | 0.11 |
|  | Ne_38 | 0.45 |
|  | Ne_8 | **0.00** |

**Table S3** χ^2^ tests of genotypic linkage disequilibrium across all pairs of loci, under the null hypothesis that genotypes at one locus are independent of genotypes of another locus.

| **Sp.** | **Locus 1** | **Locus 2** | **Chi** | **df** | **P** |
| --- | --- | --- | --- | --- | --- |
| *N. bruchi* |  |  |  |  |  |
|  | Nb_27 | Nb_26 | 114.28 | 16 | < 0.00 |
|  |  |  |  |  |  |
| *N. eichhorniae* | Ne_94 | Ne_42 | 73.36 | 22 | < 0.00 |
|  | Ne_17 | Ne_56 | 51.00 | 22 | 0.00 |
|  | Ne_14 | Ne_8 | 47.30 | 20 | 0.00 |
|  | Ne_26 | Ne_94 | 37.71 | 20 | 0.01 |
|  | Ne_14 | Ne_42 | 31.71 | 20 | 0.05 |
|  |  |  |  |  |  |

**Table S4** Frequency of null alleles based on Brookfield’s estimation methods for *N. bruchi* and *N. eichhorniae* across eight and eleven collection localities respectively.

| *N. bruchi* | Nb_40 | Nb_46 | Nb_27 | Nb_5 | Nb_43 | Nb_8 | Nb_26 | Nb_13 |  |  |
| --- | --- | --- | --- | --- | --- | --- | --- | --- | --- | --- |
| Observed frequency | 0.17 | 0.17 | 0.07 | 0.01 | 0.01 | 0.11 | 0.08 | -0.01 |  |  |
| Median frequency | 0.17 | 0.16 | 0.07 | 0.01 | 0.01 | 0.1 | 0.08 | -0.02 |  |  |
| 2.5th percentile | 0.12 | 0.11 | 0.02 | -0.01 | -0.01 | 0.06 | 0.03 | -0.04 |  |  |
| 97.5th percentile | 0.24 | 0.21 | 0.13 | 0.05 | 0.05 | 0.15 | 0.13 | 0.02 |  |  |
| *N. eichhorniae* | Ne_14 | Ne_91 | Ne_26 | Ne_94 | Ne_17 | Ne_13 | Ne_42 | Ne_56 | Ne_38 | Ne_8 |
| Observed frequency | 0.04 | 0.14 | 0.21 | 0.12 | 0.14 | 0.06 | 0.02 | 0.05 | 0.00 | 0.07 |
| Median frequency | 0.04 | 0.13 | 0.21 | 0.12 | 0.14 | 0.06 | 0.02 | 0.05 | 0.00 | 0.07 |
| 2.5th percentile | 0.01 | 0.09 | 0.16 | 0.08 | 0.10 | 0.03 | -0.01 | 0.02 | -0.01 | 0.04 |
| 97.5th percentile | 0.08 | 0.18 | 0.26 | 0.16 | 0.18 | 0.10 | 0.05 | 0.09 | 0.01 | 0.11 |

**Fig. S1** Genotype accumulation curve for *N. bruchi*, across eight collection localities and eight microsatellite loci.

**Fig. S2** Genotype accumulation curve for *N. eichhorniae*, across eleven collection localities and ten microsatellite loci.

**
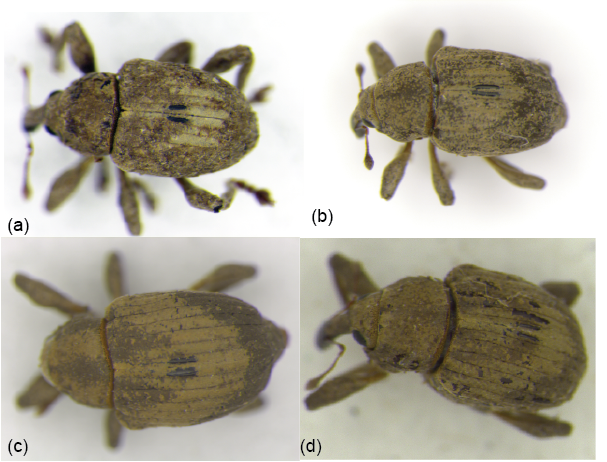
**

**Fig. S3:** Typical elytra markings characteristic of (a) *Neochetina bruchi* and (b) *N. eichhorniae;* compared to atypical elytra markings for (c) *N. bruchi* and (d) *N. eichhorniae.* Specimens (a, b, c) were collected from two study sites in the associated tributaries of the Sacramento-San Joaquin River Delta, California, and specimen (d) was collected from Wallisville, Texas. Species identifications for (a-d) were confirmed with sequences of a 616 bp fragment of the mitochondrial cytochrome oxidase subunit 1 (COI) gene (Hopper et al. 2017). However, microsatellite markers confirmed that specimens (c & d) are hybrids. A weevil (c) from the study site in California resulted in 100% amplification of markers for *N. bruchi* and 80% amplification of the markers for *N. eichhorniae*, whereas a weevil from Texas (d) resulted in amplification of 25% of the markers for *N. bruchi* and 100% of the markers for *N. eichhorniae.*

**Fig. S4:** The number of introduction steps of populations from the native range into the introduced range and (a) the allelic richness and (b) expected heterozygosity for populations of *N. bruchi* (grey markers) and *N. eichhorniae* (black markers). The number of introduced steps was calculated from the documented importation history and presented in Fig. 1 of the main article. ‘UG’ notes the points from populations in Uganda.
