# Supplementary material for: Into the weeds: Matching importation history to genetic consequences and pathways in two widely used biological control agents
Source: Evol Appl. 2019 Jan 4;12(4):773–90. doi: 10.1111/eva.12755 (PMC6439500; doi:10.1111/eva.12755)
Supplement: Supplementary file 2 [file EVA-12-773-s002.docx]

**A. PLATEAU ANALYSES AND POPULATION STRUCTURE FROM FLOCK RUNS**

* See Table 1 for full study site names and collection information

| **INITIAL POPULATION STRUCTURE ANALYSIS IN FLOCK for *N. bruchi* (NB)** | | | | | | | |  | |  | | | |  |
| --- | --- | --- | --- | --- | --- | --- | --- | --- | --- | --- | --- | --- | --- | --- |
| **k** | | **Completed runs** | | **Aborted runs** | | **Sequence of plateau lengths** | | | | | | | |  |
| 2 | | 50 | | 0 | | 3, 2, 3, 2, 2, 2, 2 | | | |  | | | |  |
| 3 | | 50 | | 0 | | 2 | |  | |  | | | |  |
| 4 | | 50 | | 0 | | 0 | |  | |  | | | |  |
| 5 | | 50 | | 0 | | 0 | |  | |  | | | |  |
| 6 | | 50 | | 0 | | 0 | |  | |  | | | |  |
| 7 | | 50 | | 0 | | 0 | |  | |  | | | |  |
|  | | SPECIMENS ALLOCATED | | | |  | |  | |  | | | |  |
| **Population** | | | **ref1** | **ref2** | |  | | **MLLOD = 2.50** | | | |  | |  |
| **NB AU** (Australia) | | | 3 | 18 | |  | |  | |  | | | |  |
| **NB CA** (USA: California) | | | 7 | 18 | |  | |  | |  | | | |  |
| **NB FL** (USA: Florida) | | | 1 | 20 | |  | |  | |  | | | |  |
| **NB SAW** (SA: Wolseley) | | | 1 | 5 | |  | |  | |  | | | |  |
| **NB SAE** (SA: Enseleni) | | | 9 | 9 | | SAE IS A COMPOSITE POPULATION | | | | | | | |  |
| **NB TX** (USA: Texas) | | | 24 | 1 | |  | |  | |  | | | |  |
| **NB UG** (Uganda) | | | 2 | 24 | |  | |  | |  | | | |  |
| **NB UR** (Uruguay) | | | 21 | 8 | |  | |  | |  | | | |  |
|  | | | | | | | | | |  | | | |  |
| **INITIAL POPULATION STRUCTURE ANALYSIS IN FLOCK WITHOUT SAE** | | | | | | | | | |  | | | |  |
| k | | Completed runs | | Aborted runs | | Sequence of plateau lengths | | | | | | | |  |
| 2 | | 50 | | 0 | | 5, 4, 12, 4, 2, 2, 2, 2, 2 | | | | | | |  |  |
| 3 | | 50 | | 0 | | 2, 2 | |  |  | | | | | |
| 4 | | 50 | | 0 | | 2 | |  |  | | | | | |
| 5 | | 50 | | 0 | | 0 | |  |  | | | | | |
| 6 | | 50 | | 0 | | 0 | |  |  | | | | | |
| 7 | | 50 | | 0 | | 0 | |  |  | | | | | |
| 8 | | 50 | | 0 | | 0 | |  |  | | | | | |
|  | | SPECIMENS ALLOCATED | | | |  | |  | | | |  | |  |
| **Populations** | | **ref1** | | **ref2** | |  | | **MLLOD = 2.58** | | | |  | |  |
| **NB AU** | | 20 | | 1 | |  | |  | | | |  | |  |
| **NB CA** | | 14 | | 11 | | CA IS A COMPOSITE POPULATION | | | | | | | |  |
| **NB FL** | | 19 | | 2 | |  | |  | |  | | | |  |
| **NB SAW** | | 5 | | 1 | |  | |  | |  | | | |  |
| **NB TX** | | 0 | | 25 | |  | |  | |  | | | |  |
| **NB UG** | | 21 | | 5 | |  | |  | |  | | | |  |
| **NB UR** | | 4 | | 25 | |  | |  | |  | | | |  |
|  | | | | | | | |  | |  | | | |  |
|  | | | | | | | |  | |  | | | |  |
|  | | | | | | | |  | |  | | | |  |
|  | | | | | | | |  | |  | | | |  |
|  | | | | | | | |  | |  | | | |  |
| **POPULATION STRUCTURE in FLOCK without CA or SAE** | | | | | | | |  | |  | | | |  |
| k | Completed runs | | | | Aborted runs | | Sequence of plateau lengths | | | | | | |  |
| 2 | 50 | | | | 0 | | 10, 3, 3, 2, 3, 2, 2, 3, 4, 3 | | | | | | |  |
| 3 | 50 | | | | 0 | | 2, 2 | | | | | | |  |
| 4 | 50 | | | | 0 | | 2 | | | | | | |  |
| 5 | 50 | | | | 0 | | 0 | | | | | | |  |
| 6 | 50 | | | | 0 | | 0 | | | | | | |  |
| 7 | 50 | | | | 0 | | 0 | | | | | | |  |
| 8 | 47 | | | | 3 | | 0 | | | | | | |  |
|  | | SPECIMENS ALLOCATED | | | |  | |  | | |  | | |  |
| **Populations** | | **ref1** | | **ref2** | |  | | **MLLOD = 2.69** | | |  | | |  |
| **NB AU** | | 20 | | 1 | |  | |  | | |  | | |  |
| **NB FL** | | 19 | | 2 | |  | |  | | |  | | |  |
| **NB SAW** | | 6 | | 0 | |  | |  | | |  | | |  |
| **NB TX** | | 0 | | 25 | |  | |  | | |  | | |  |
| **NB UG** | | 24 | | 2 | |  | |  | | |  | | |  |
| **NB UR** | | 7 | | 22 | |  | |  | | |  | | |  |
| **POPULATION STRUCTURE OF AU,FL,SAW,UG** | | | | | |  | |  | | |  | | |  |
| **k is undecided (k = 1)** | | **plateaus: 2, 3, 4, 2** | | | |  | |  | | |  | | |  |
| **Mean LLOD** | | **1.735295266** | |  | |  | |  | | |  | | |  |
|  | | SPECIMENS ALLOCATED | | | |  | |  | | |  | | |  |
| **Populations** | | **ref1** | | **ref2** | | **Note: output for k =2** | | | | |  | | |  |
| **NB AU** | | 12 | | 9 | | Results indicate that max population | | | | | | | |  |
| **NB FL** | | 12 | | 9 | | structure has been reached at this point | | | | | | | |  |
| **NB SAW** | | 2 | | 4 | |  | |  | |  | | | |  |
| **NB UG** | | 10 | | 16 | |  | |  | |  | | | |  |
|  | |  | |  | |  | |  | |  | | | |  |

**B. FLOCK plateau analysis for population substructure analyses**

|  | **Population substructure** | | | |  | |  |  | |  |
| --- | --- | --- | --- | --- | --- | --- | --- | --- | --- | --- |
| **K for TX** | **Completed runs** | **Aborted runs** | | | **Sequence of plateau lengths** | | | | |  |
| 2 | 50 | 0 | | | 18, 26 | |  |  | |  |
| 3 | 49 | 1 | | | 16, 8, 6, 2 | | | | |  |
| 4 | 43 | 7 | | | 2, 2, 5, 2, 2, 3, 2, 2 | | |  | |  |
| 5 | 18 | 32 | | | 2, 3, 2 | |  |  | |  |
| 6 | 1 | 49 | | | 0 | |  |  | |  |
| **K = 3** | **SPECIMENS ALLOCATED** | | | |  | |  |  | |  |
|  | **ref1** | **ref2** | | | **ref3** | | **Mean LLOD** | **2.21** | |  |
| **NB TX** | 9 | 9 | | | 7 | |  |  | |  |
|  |  |  | | |  | |  |  | |  |
| **K for CA** | **Completed runs** | **Aborted runs** | | | **Sequence of plateau lengths** | | | | |  |
| 2 | 50 | 0 | | | 35, 6 | |  | | |  |
| 3 | 50 | 0 | | | 2, 2, 3, 2, 3, 3, 2, 2 | | |  | |  |
| 4 | 14 | 36 | | | 0 | |  |  | |  |
| 5 | 2 | 48 | | | 0 | |  |  | |  |
| 6 | 0 | 50 | | | 0 | |  |  | |  |
| **K = 2** | **SPECIMENS ALLOCATED** | | | |  | |  |  | |  |
|  | **ref1** | **ref2** | | |  | | **Mean LLOD** | **2.67** | |  |
| **NB CA** | 12 | 13 | | |  | |  |  | |  |
|  |  |  | | |  | |  |  | |  |
| **K for UR** | **Completed runs** | | **Aborted runs** | | **Sequence of plateau lengths** | | | | |  |
| 2 | 50 | 0 | | | 9, 8, 15, 2 | | |  | |  |
| 3 | 35 | 15 | | | 4, 5, 3 | |  |  | |  |
| 4 | 12 | 38 | | | 0 | |  |  | |  |
| 5 | 2 | 48 | | | 0 | |  |  | |  |
| 6 | 0 | 50 | | | 0 | |  |  | |  |
|  |  | | | |  | |  |  | |  |
|  |  | | | |  | |  |  | |  |
| **K = 2** | **SPECIMENS ALLOCATED** | | | |  | |  |  | |  |
|  | **ref1** | **ref2** | | |  | | **Mean LLOD** | **2.01** | |  |
| **NB UR** | 14 | 15 | | |  | |  |  | |  |
|  |  |  | | |  | |  |  | |  |
| **K for SAE** | **Completed runs** | | | **Aborted runs** | | **Sequence of plateau lengths** | | | |  |
| 2 | 50 | 0 | | | 2, 4, 9, 2, 12, 8, 4, 2 | | |  |  |  |
| 3 | 40 | 10 | | | 3, 4, 6, 4, 3, 4, 3 | | |  |  |  |
| 4 | 2 | 48 | | | 0 | | |  |  |  |
| **K = 2** | **SPECIMENS ALLOCATED** | | | |  | |  |  | |  |
|  | **ref1** | **ref2** | | |  | | **Mean LLOD** | **3.12** | |  |
| **NB SAE** | **10** | **8** | | |  | |  |  | |  |
|  |  |  | | |  | |  |  | |  |
| **K for FL** | **Completed runs** | **Aborted runs** | | | **Sequence of plateau lengths** | | | | |  |
| 2 | 50 | 0 | | | 42, 3 | | | | |  |
| 3 | 46 | 4 | | | 2, 4, 3 | | | | |  |
| 4 | 27 | 23 | | | 2 | | | | |  |
| 5 | 3 | 47 | | | 0 | | | | |  |
| 6 | 0 | 50 | | | 0 | | | | |  |
| **K = 2** | **SPECIMENS ALLOCATED** | | | |  | |  |  | |  |
|  | **ref1** | **ref2** | | |  | | **Mean LLOD** | **2.32** | |  |
| **NB FL** | **9** | **12** | | |  | |  |  | |  |
|  |  |  | | |  | |  |  | |  |

**C. FLOCK ALLOCATION TABLES FOR EACH POPULATION**

| **FL Analysis** | |  | | |  | | | | | **MLLOD** | | | | | | **P-value** | | | | | | |  | | | | |  |  |  |  |  |  |
| --- | --- | --- | --- | --- | --- | --- | --- | --- | --- | --- | --- | --- | --- | --- | --- | --- | --- | --- | --- | --- | --- | --- | --- | --- | --- | --- | --- | --- | --- | --- | --- | --- | --- |
|  | | **ref1** | | | **ref2** | | | | | **3.20** | | | | | | **< 0.001** | | | | | | |  | | | | |  |  |  |  |  |  |
| **NBFL** | | **19** | | | **2** | | | | |  | | | | | |  | | | | | | |  | | | | |  |  |  |  |  |  |
| **UR1** | | **7** | | | **7** | | | | |  | | | | | |  | | | | | | |  | | | | |  |  |  |  |  |  |
| **UR2** | | 0 | | | **15** | | | | |  | | | | | |  | | | | | | |  | | | | |  |  |  |  |  |  |
| \| **UR1 is the most likely source of FL** \|  \|  \| \| --- \| --- \| --- \| | | | | | | | | | | | | | | | | | | | | | | | | | | | |  |  |  |  |  |  |
| **AU Analysis 1** | |  | | |  | | | | | **MLLOD** | | | | | | **P-value** | | | | | | |  | | | | |  |  |  |  |  |  |
|  | | **ref1** | | | **ref2** | | | | | **2.65** | | | | | | **< 0.001** | | | | | | |  | | | | |  |  |  |  |  |  |
| **NB AU** | | **19** | | | **2** | | | | |  | | | | | |  | | | | | | |  | | | | |  |  |  |  |  |  |
| **NB FL** | | **19** | | | **2** | | | | |  | | | | | |  | | | | | | |  | | | | |  |  |  |  |  |  |
| **NB UR1** | | 4 | | | **10** | | | | |  | | | | | |  | | | | | | |  | | | | |  |  |  |  |  |  |
| **NB UR2** | | 0 | | | **15** | | | | |  | | | | | |  | | | | | | |  | | | | |  |  |  |  |  |  |
| **FL is the most likely source of AU** | | | | | | | | | | | | | |  | | | | | | | |  | | | | | | | |  |  |  |  |
| **NB CA1 Analysis 1** | | | | | | | **MLLOD** | | | | | | | **P-value** | | | | | | | | | | |  | | | | |  |  |  |  |
|  | **ref1** | | **ref2** | | | | **2.49** | | | | | | | **< 0.001** | | | | | | | | | | |  | | | | |  |  |  |  |
| **NB CA1** | **1** | | **11** | | | |  | | | | | | |  | | | | | | | | | | |  | | | | |  |  |  |  |
| **NB FL** | 2 | | **19** | | | |  | | | | | | |  | | | | | | | |  | | | | | | | |  |  |  |  |
| **NB TX1** | 2 | | **7** | | | |  | | | | | | |  | | | | | | | |  | | | | | | | |  |  |  |  |
| **NB TX2** | **7** | | 0 | | | |  | | | | | | |  | | | | | | | |  | | | | | | | |  |  |  |  |
| **NB TX3** | **6** | | 3 | | | |  | | | | | | |  | | | | | | | |  | | | | | | | |  |  |  |  |
| **NB UR1** | **10** | | 4 | | | |  | | | | | | |  | | | | | | | |  | | | | | | | |  |  |  |  |
| **NB UR2** | **15** | | 0 | | | |  | | | | | | |  | | | | | | | |  | | | | | | | |  |  |  |  |
| **NB CA1 Analysis 2** | | | | | | | **MLLOD** | | | | | | | **P-value** | | | | | | | | | | | |  | | | |  |  |  |  |
|  | **ref1** | | **ref2** | | | | **4.14** | | | | | | | **< 0.001** | | | | | | | | | | | |  | | | |  |  |  |  |
| **NB CA1** | 0 | | 12 | | | |  | | | | | | |  | | | | | | | | | | | |  | | | |  |  |  |  |
| **NB FL** | 1 | | **20** | | | |  | | | | | | |  | | | | | | | |  | | | | | | | |  |  |  |  |
| **NB TX1** | **9** | | 0 | | | |  | | | | | | |  | | | | | | | |  | | | | | | | |  |  |  |  |
| **FL is the most likely source of NB CA1** | | | | | | | | | | | | | |  | | | | | | | |  | | | | | | | |  |  |  |  |
| **NB CA2 Analysis 1** | | | | | | | | | | | | **MLLOD** | | | | | **P-value** | | | | | | | | | |  |  |  |  |  |  |  |
|  | | **ref1** | | | | | | **ref2** | | | | **3.22** | | | | | **< 0.001** | | | | | | | | | |  |  |  |  |  |  |  |
| **NB CA2** | | 3 | | | | | | 10 | | | |  | | | | |  | | | | | | | | | |  |  |  |  |  |  |  |
| **NB FL** | | 2 | | | | | | **19** | | | |  | | | | |  | | | | | | | | | |  |  |  |  |  |  |  |
| **NB TX1** | | 0 | | | | | | **9** | | | |  | | | | |  | | | | | | | | | |  |  |  |  |  |  |  |
| **NB TX2** | | **7** | | | | | | 0 | | | |  | | | | |  | | | | | | | | | |  |  |  |  |  |  |  |
| **NB TX3** | | 0 | | | | | | **9** | | | |  | | | | |  | | | | | | | | | |  |  |  |  |  |  |  |
| **NB UR1** | | **8** | | | | | | 6 | | | |  | | | | |  | | | | | | | | | |  |  |  |  |  |  |  |
| **NB UR2** | | **14** | | | | | | 1 | | | |  | | | | |  | | | | | | | | | |  |  |  |  |  |  |  |
|  | | | | | | | | | | | |  | | | | |  | | | | | | | | | |  |  |  |  |  |  |  |
|  | | | | | | | | | | | |  | | | | |  | | | | | | | | | |  |  |  |  |  |  |  |
|  | | | | | | | | | | | |  | | | | |  | | | | | | | | | |  |  |  |  |  |  |  |
|  | | | | | | | | | | | |  | | | | |  | | | | | | | | | |  |  |  |  |  |  |  |
|  | | | | | | | | | | | |  | | | | |  | | | | | | | | | |  |  |  |  |  |  |  |
| **C. FLOCK ALLOCATION TABLES FOR EACH POPULATION- Continued** | | | | | | | | | | | | | | | | | | | | | | | | | | | |  |  |  |  |  |  |
| **NB CA2 Analysis** | | | | | | | | | | | | **MLLOD** | | | | | **P-value** | | | | | | | | | |  |  |  |  |  |  |  |
|  | | **ref1** | | | | | | **ref2** | | | | **2.92** | | | | | **< 0.001** | | | | | | | | | |  |  |  |  |  |  |  |
| **NB CA2** | | 7 | | | | | | 6 | | | |  | | | | |  | | | | | | | | | |  |  |  |  |  |  |  |
| **NB FL** | | 1 | | | | | | 20 | | | |  | | | | |  | | | | | | | | | |  |  |  |  |  |  |  |
| **NB TX1** | | 9 | | | | | | 0 | | | |  | | | | |  | | | | | | | | | |  |  |  |  |  |  |  |
| **NB TX3** | | 8 | | | | | | 1 | | | |  | | | | |  | | | | | | | | | |  |  |  |  |  |  |  |
| **FL, TX1, TX3 are likely sources of CA2**  ***See supplementary Appendix 5 for further clarification with DAPC*** | | | | | | | | | | | | | | | | | | | | | | | | | | |  |  |  |  |  |  |  |
| **SAW, Analysis 1** | | | | |  | | | | | | | **MLLOD** | | | | | **P-value** | | | | | |  | | | | |  | | | | | |
|  | | | **ref1** | | | | | **ref2** | | | | **2.69** | | | **< 0.001** | | | | | | | | | | | | |  | | | | | |
| **NB SAW** | | | 1 | | | | | 5 | | | |  | | | | | | | | | | |  | | | | |  | | | | | |
| **NB AU** | | | 2 | | | | | 19 | | | |  | | | | | | | | | | |  | | | | |  | | | | | |
| **NB FL** | | | 2 | | | | | 19 | | | |  | | | | | | | | | | |  | | | | |  | | | | | |
| **NB UR1** | | | **11** | | | | | 3 | | | |  | | | | | | | | | | |  | | | | |  | | | | | |
| **NB UR2** | | | **15** | | | | | 0 | | | |  | | | | | | | | | | |  | | | | |  | | | | | |
| **SAW Analysis 2** | | |  |  | | | | | **MLLOD** | | | | | | | | | | | **P-value** | | | | | | | | | | |  |  |  |
|  | | | **ref1** | | | | | **ref2** | | | | | **1.97** | | **0.29** | | | | | | | |  |  |  |  |  |  |  |  |  |  |  |
| **NB SAW** | | | 3 | | | | | 3 | | | | |  | | | | | | | | | |  | | | | |  |  |  |  |  |  |
| **NB AU** | | | 12 | | | | | 9 | | | | |  | | | | | | | | | |  | | | | |  |  |  |  |  |  |
| **NB FL** | | | 7 | | | | | 14 | | | | |  | | | | | | | | | |  | | | | |  |  |  |  |  |  |
| **AU and FL are the most likely sources of SAW** | | | | | | | | | | | | | | | | | | | | | | | | | | | |  |  |  |  |  |  |
| **SAE1, Analysis 1** | | | | | | | |  | | | | **MLLOD** | | | | | | **P-value** | | |  | | | | | | |  |  |  |  |  |  |
|  | | | **ref1** | | | | | **ref2** | | | | **2.42** | | | | | | **< 0.001** | | |  | | | | | | |  |  |  |  |  |  |
| **NB SAE1** | | | **1** | | | | | **9** | | | |  | | | | | |  | | |  | | | | | | |  |  |  |  |  |  |
| **NB AU** | | | **18** | | | | | 3 | | | |  | | | | | |  | | |  | | | | | | |  |  |  |  |  |  |
| **NB FL** | | | **19** | | | | | 2 | | | |  | | | | | |  | | |  | | | | | | |  |  |  |  |  |  |
| **NB UR1** | | | 4 | | | | | **10** | | | |  | | | | | |  | | |  | | | | | | |  |  |  |  |  |  |
| **NB UR2** | | | 0 | | | | | **15** | | | |  | | | | | |  | | |  | | | | | | |  |  |  |  |  |  |
| **SAE1, Analysis 2** | | | | | | | |  | | | | **MLLOD** | | | | | | **P-value** | | |  | | | | | | |  |  |  |  |  |  |
|  | | | **ref1** | | | | | **ref2** | | | | **3.06** | | | | | | **< 0.001** | | |  | | | | | | |  |  |  |  |  |  |
| **NB SAE1** | | | **9** | | | | | **1** | | | |  | | | | | |  | | |  | | | | | | |  |  |  |  |  |  |
| **NB UR1** | | | 4 | | | | | 10 | | | |  | | | | | |  | | |  | | | | | | |  |  |  |  |  |  |
| **NB UR2** | | | 2 | | | | | 13 | | | |  | | | | | |  | | |  | | | | | | |  |  |  |  |  |  |
| **NB UR1 is the most likely source of SAE1, *other sources likely exist** | | | | | | | | | | | | | | | | | | | | | | | | | | | |  |  |  |  |  |  |
| **SAE2, Analysis 1** | | |  | | | | |  | | | | **MLLOD** | | | | | **P-value** | | | | |  | | | | | |  |  |  |  |  |  |
|  | | | **ref1** | | | | | **ref2** | | | | **3.06** | | | | | **< 0.001** | | | | |  | | | | | |  |  |  |  |  |  |
| **NB SAE2** | | | **0** | | | | | **8** | | | |  | | | | |  | | | | |  | | | | | |  |  |  |  |  |  |
| **NB AU** | | | 8 | | | | | 13 | | | |  | | | | |  | | | | |  | | | | | |  |  |  |  |  |  |
| **NB FL1** | | | 0 | | | | | 9 | | | |  | | | | |  | | | | |  | | | | | |  |  |  |  |  |  |
| **NB FL2** | | | 3 | | | | | 9 | | | |  | | | | |  | | | | |  | | | | | |  |  |  |  |  |  |
| **NB UR1** | | | **8** | | | | | 6 | | | |  | | | | |  | | | | |  | | | | | |  |  |  |  |  |  |
| **NB UR2** | | | **14** | | | | | 1 | | | |  | | | | |  | | | | |  | | | | | |  |  |  |  |  |  |
| **C. FLOCK ALLOCATION TABLES FOR EACH POPULATION- Continued** | | | | | | | | | | | | | | | | | | | | | | | | | | | |  |  |  |  |  |  |
| **SAE2, Analysis 2** | | |  | | | | |  | | | | **MLLOD** | | | | | **P-value** | | | | |  | | | | | |  |  |  |  |  |  |
|  | | | **ref1** | | | | | **ref2** | | | | **2.01** | | | | | **< 0.001** | | | | |  | | | | | |  |  |  |  |  |  |
| **NB SAE2** | | | **6** | | | | | **2** | | | |  | | | | |  | | | | |  | | | | | |  |  |  |  |  |  |
| **NB AU** | | | **10** | | | | | **11** | | | |  | | | | |  | | | | |  | | | | | |  |  |  |  |  |  |
| **NB FL1** | | | 9 | | | | | 0 | | | |  | | | | |  | | | | |  | | | | | |  |  |  |  |  |  |
| **NB FL2** | | | **0** | | | | | **12** | | | |  | | | | |  | | | | |  | | | | | |  |  |  |  |  |  |
| **SAE2, Analysis 3** | | |  | | | | |  | | | | **MLLOD** | | | | | **P-value** | | | | |  | | | | | |  |  |  |  |  |  |
|  | | | **ref1** | | | | | **ref2** | | | | **2.18** | | | | | **0.82** | | | | |  | | | | | |  |  |  |  |  |  |
| **NB SAE2** | | | **4** | | | | | **4** | | | |  | | | | |  | | | | |  | | | | | |  |  |  |  |  |  |
| **NB FL1** | | | 5 | | | | | 4 | | | |  | | | | |  | | | | |  | | | | | |  |  |  |  |  |  |
| \| **FL1 is the most likely source of SAE2** \|  \| \| --- \| --- \| | | | | | | | | | | | | | | | | | | | | | | | | | | | |  |  |  |  |  |  |
|  | | |  | | |  | | | | | **MLLOD** | | | | | | **P-value** | | | | | |  | | | | |  |  |  |  |  |  |
|  | | | **ref1** | | | **ref2** | | | | | **2.97** | | | | | | **< 0.001** | | | | | |  | | | | |  |  |  |  |  |  |
| **NB TX1** | | | 0 | | | 9 | | | | |  | | | | | |  | | | | | |  | | | | |  |  |  |  |  |  |
| **NB FL** | | | 3 | | | **18** | | | | |  | | | | | |  | | | | | |  | | | | |  |  |  |  |  |  |
| **NB UR1** | | | **11** | | | 3 | | | | |  | | | | | |  | | | | | |  | | | | |  |  |  |  |  |  |
| **NB UR2** | | | **15** | | | 0 | | | | |  | | | | | |  | | | | | |  | | | | |  |  |  |  |  |  |
| **FL is the most likely source of NB TX1** | | | | | | | | | | | | | | | | | | | | | | |  | | | | |  |  |  |  |  |  |
|  | | |  | | |  | | | | | **MLLOD** | | | | | | **P-VALUE** | | | | | |  | | | | |  |  |  |  |  |  |
|  | | | **ref1** | | | **ref2** | | | | | **3.35** | | | | | | **< 0.001** | | | | | |  | | | | |  |  |  |  |  |  |
| **NB TX2** | | | **7** | | | **0** | | | | |  | | | | | |  | | | | | |  | | | | |  |  |  |  |  |  |
| NB FL | | | 2 | | | **19** | | | | |  | | | | | |  | | | | | |  | | | | |  |  |  |  |  |  |
| **NB UR1** | | | 7 | | | **7** | | | | |  | | | | | |  | | | | | |  | | | | |  |  |  |  |  |  |
| **NB UR2** | | | **15** | | | 0 | | | | |  | | | | | |  | | | | | |  | | | | |  |  |  |  |  |  |
| **NB UR2 is the most likely source of NB TX2** | | | | | | | | | | | | | | | | | | | | | | |  | | | | |  |  |  |  |  |  |
| **TX 3** | | |  | | |  | | | | **MLLOD** | | | | | | **P-value** | | | | | | |  | | | | |  |  |  |  |  |  |
|  | | | **ref1** | | | **ref2** | | | | | **3.15** | | | | | **1.44E-07** | | | | | | |  | | | | |  |  |  |  |  |  |
| **NB TX3** | | | 9 | | | 0 | | | |  | | | | | |  | | | | | | |  | | | | |  |  |  |  |  |  |
| **NB FL** | | | **18** | | | 3 | | | |  | | | | | |  | | | | | | |  | | | | |  |  |  |  |  |  |
| **NB UR1** | | | 6 | | | **8** | | | |  | | | | | |  | | | | | | |  | | | | |  |  |  |  |  |  |
| **NB UR2** | | | 0 | | | **15** | | | |  | | | | | |  | | | | | | |  | | | | |  |  |  |  |  |  |
| **FL is the most likely source of NB TX3** | | | | | | | | | | | | | | | | | | | | | | |  | | | | |  |  |  |  |  |  |
| **UG, Analysis 1** | | |  | | | | |  | | | | **MLLOD** | | | | | | | **P-value** | | | | | | | | | |  | | |  |  |
|  | | | **ref1** | | | | | **ref2** | | | | **2.45** | | | | | | | **< 0.001** | | | | |  | | | | | | | |  |  |
| **NB UG** | | | **21** | | | | | **5** | | | |  | | | | | | |  | | | | |  | | | | | | | |  |  |
| **NB AU** | | | 19 | | | | | 2 | | | |  | | | | | | |  | | | | |  | | | | | | | |  |  |
| **NB FL** | | | 19 | | | | | 2 | | | |  | | | | | | |  | | | | |  | | | | | | | |  |  |
| **NB UR1** | | | 3 | | | | | **11** | | | |  | | | | | | |  | | | | |  | | | | | | | |  |  |
| **NB UR2** | | | 0 | | | | | **15** | | | |  | | | | | | |  | | | | |  |  |  |  |  |  |  |  |  |  |
|  | | |  | | | | |  | | | |  | | | | | | |  | | | | |  |  |  |  |  |  |  |  |  |  |
|  | | |  | | | | |  | | | |  | | | | | | |  | | | | |  |  |  |  |  |  |  |  |  |  |
| **UG, Analysis 2** | | |  | | | | |  | | | | **MLLOD** | | | | | | | **P-value** | | | | | | | | | | | | | |  |
|  | | | **ref1** | | | | | **ref2** | | | | **1.93** | | | | | | | **< 0.001** | | | | | | | | | | | | | |  |
| **NB UG** | | | **14** | | | | | **12** | | | |  | | | | | | |  | | | | | | | | | | | | | |  |
| **NB AU** | | | 12 | | | | | 9 | | | |  | | | | | | |  | | | | | | | | | | | | | |  |
| **NB FL** | | | 11 | | | | | 10 | | | |  | | | | | | |  | | | | | | | | | | | | | |  |
| **AU and FL are the most likely sources of UG** | | | | | | | | | | | | | | | | | | | | | | | | | | | | | | | | |  |

**Additional DAPC for clarification of FLOCK ALLOCATION TABLES**

Using the FLOCK allocation tables, we visualized the different potential clusters with DAPC to determine the potential genetic sources when FLOCK results were not clear.

| **NB CA2 Analysis 1** |  |  | **MLLOD** | **P-value** |  |
| --- | --- | --- | --- | --- | --- |
|  | **ref1** | **ref2** | **3.22** | **< 0.001** |  |
| **NB CA2** | 3 | 10 |  |  |  |
| **NB FL** | 2 | **19** |  |  |  |
| **NB TX1** | 0 | **9** |  |  |  |
| **NB TX2** | **7** | 0 |  |  |  |
| **NB TX3** | 0 | **9** |  |  |  |
| **NB UR1** | **8** | 6 |  |  |  |
| **NB UR2** | **14** | 1 |  |  |  |
| **NB CA2 Analysis 2** |  |  | **MLLOD** | **P-value** |  |
|  | **ref1** | **ref2** | **2.92** | **< 0.001** |  |
| **NB CA2** | 7 | 6 |  |  |  |
| **NB FL** | 1 | 20 |  |  |  |
| **NB TX1** | 9 | 0 |  |  |  |
| **NB TX3** | 8 | 1 |  |  |  |
| **FL, TX1, TX3 are likely sources of NB CA2 based on FLOCK**  **DAPC BELOW SUGGESTS FL, TX1, UR1 and UR2 are more likely sources of CA2** | | | | |  |
| **** | | | | | |

**FLOCK SEARCHING PROCEDURE**

Searching procedure for the most likely source S of colony C among putative candidates {Pi}

1. FLOCK (k = 2) C with all (remaining) candidate sources {Pi}
2. **If** there are *unlikely* sources

a) remove *unlikely* sources from candidate sources

b) go to 1)

**else**

if only one remaining candidate source Pk

then S = Pk i.e. it is the most likely source among candidate sources

if more than one remaining putative source then

for each remaining i , FLOCK (k = 2) all C and Pi pairs

compare outputs and keep the putative source(s) that are least differentiated from C (from allocation tables)

*unlikely*: if C has been mainly allocated to A/B and P has been mainly allocated to B/A (the alternative cluster) then P is considered *unlikely*

***Example***

|  | Allocated to | |
| --- | --- | --- |
| Number of specimens among | **ref1** | **ref2** |
| **Pop1** | **4** | **19** |
| **Pop2** | 1 | 19 |
| **Pop3** | **23** | **0** |
| **Pop4** | 2 | 19 |
| **Pop5** | 0 | 16 |
| **Pop6** | 3 | 12 |
|  |  |  |
| Number of specimens among | **ref1** | **ref2** |
| **Pop1** | **23** | **0** |
| **Pop2** | 16 | 4 |
| **Pop4** | 17 | 4 |
| **Pop5** | **0** | **16** |
| **Pop6** | **7** | **8** |
|  |  |  |
| Number of specimens among | **ref1** | **ref2** |
| **Pop1** | **8** | **15** |
| **Pop2** | **18** | **2** |
| **Pop4** | 7 | 14 |
|  |  |  |

**Pop4 is the most likely source of Pop1**
