# Supplementary material for: Into the weeds: Matching importation history to genetic consequences and pathways in two widely used biological control agents
Source: Evol Appl. 2019 Jan 4;12(4):773–90. doi: 10.1111/eva.12755 (PMC6439500; doi:10.1111/eva.12755)

**A. PLATEAU ANALYSES AND POPULATION STRUCTURE FROM FLOCK RUNS**

* See Table 1 for full study site names and collection information

| **INITIAL POPULATION STRUCTURE ANALYSIS IN FLOCK for *N. eichhorniae* (NE)** | | | |  |  |
| --- | --- | --- | --- | --- | --- |
| **k** | **Completed runs** | **Aborted runs** | **Sequence of plateau lengths** | | |

| 2 | 50 | 0 | 10, 2, 2, 21, 2 |
| --- | --- | --- | --- |
| 3 | 50 | 0 | 3, 2, 3, 2, 2 |
| 4 | 50 | 0 | 0 |
| 5 | 50 | 0 | 0 |
| 6 | 50 | 0 | 0 |

| **K = 2** | | | | | | SPECIMENS ALLOCATED | | | | | **MLLOD** | | | | **P-VALUE** | |  | |  |  |  |
| --- | --- | --- | --- | --- | --- | --- | --- | --- | --- | --- | --- | --- | --- | --- | --- | --- | --- | --- | --- | --- | --- |
| Population | | | | | | **ref1** | | | | **ref2** | **3.59** | | | | **< 0.001** | |  | |  |  |  |
| **NE SAG** (SA: George) | | | | | | 22 | | | | 0 |  | | | |  | |  | |  |  |  |
| **NE AU** (Australia) | | | | | | 16 | | | | 4 |  | | | |  | |  | |  |  |  |
| **NE CH** (China) | | | | | | 1 | | | | 22 |  | | | |  | |  | |  |  |  |
| **NE CA** (USA: California) | | | | | | 24 | | | | 0 |  | | | |  | |  | |  |  |  |
| **NE FL** (USA: Florida) | | | | | | 19 | | | | 2 |  | | | |  | |  | |  |  |  |
| **NE SAW** (SA: Wolseley) | | | | | | 20 | | | | 3 |  | | | |  | |  | |  |  |  |
| **NE SAK** (SA: Kubusi) | | | | | | 18 | | | | 4 |  | | | |  | |  | |  |  |  |
| **NE SI** (Singapore) | | | | | | 1 | | | | 32 |  | | | |  | |  | |  |  |  |
| **NE TX** (USA: Texas) | | | | | | 20 | | | | 2 |  | | | |  | |  | |  |  |  |
| **NE UG** (Uganda) | | | | | | 22 | | | | 4 |  | | | |  | |  | |  |  |  |
| **NE UR** (Uruguay) | | | | | | **28** | | | | **3** |  | | | |  | |  | |  |  |  |
| **INITIAL POPULATION STRUCTURE ANALYSIS IN FLOCK WITHOUT CH AND SI** | | | | | | | | | | | | | | | | | | |  |  |  |
| k | | | | | **Completed runs** | | | | | **Aborted runs** | | | **Sequence of plateau lengths** | | | | | |  |  |  |
| 2 | | | | | 50 | | | | | 0 | 3, 2, 2 | | | | | | | |  | |  |
| 3 | | | | | 50 | | | | | 0 | 0 | | | | | | | |  | |  |
| 4 | | | | | 50 | | | | | 0 | 0 | | | | | | | |  | |  |
| 5 | | | | | 50 | | | | | 0 | 0 | | | | | | | |  | |  |
| 6 | | | | | 50 | | | | | 0 | 0 | | | | | | | |  | |  |
| **K undecided (K = 1)** | | | | **SPECIMENS ALLOCATED** | | | | | | | **MLLOD** | | |  | |  | |  |  |  |  |
| **Populations** | | | | **ref1** | | | | **ref2** | | | **2.482** | | |  | |  | |  |  |  |  |
| **NE SAG** | | | | 22 | | | | 0 | | |  | | |  | |  | |  |  |  |  |
| **NE AU** | | | | 10 | | | | 10 | | | *AU IS COMPOSITE POPULATION* | | | | | | |  |  |  |  |
| **NE CA** | | | | 8 | | | | 16 | | |  | | |  | |  | |  |  |  |  |
| **NE FL** | | | | 4 | | | | 17 | | |  | | |  | |  | |  |  |  |  |
| **NE SAW** | | | | 2 | | | | 21 | | |  | | |  | |  | |  |  |  |  |
| **NE SAK** | | | | 7 | | | | 15 | | |  | | |  | |  | |  |  |  |  |
| **NE TX** | | | | 6 | | | | 16 | | |  | | |  | |  | |  |  |  |  |
| **NE UG** | | | | 3 | | | | 23 | | |  | | |  | |  | |  |  |  |  |
| **NE UR** | | | | 24 | | | | 7 | | |  | | |  | |  | |  |  |  |  |
| **POPULATION STRUCTURE ANALYSIS OF CH AND SI ONLLY** | | | | | | | | | | | | | | | | | |  |  |  |  |
| **k** | | **Completed runs** | | | | | | **Aborted runs** | | | **Sequence of plateau lengths** | | | | | |  |  |  |  |  |
| 2 | | 50 | | | | | | | 0 | | | 50 | | | | | |  |  |  |  |
| 3 | | 50 | | | | | | | 0 | | | 2, 2, 5, 3, 2, 2, 2, 4 | | | | | | | |  |  |
| **K = 2** | | | | **SPECIMENS ALLOCATED** | | | | | | |  | **MLLOD P-VALUE** | | | | | |  |  |  |  |
| Populations | | | | **ref1** | | | **ref2** | | | |  | **5.22 <0.001** | | | | | |  |  |  |  |
| **NE CH** | | | | **0** | | | **23** | | | |  |  | | | | | |  |  |  |  |
| **NE SI** | | | | **30** | | | **3** | | | |  |  | | | | | |  |  |  |  |

| **B. FLOCK plateau analysis for population substructure analyses** | | | | | | | | | | | | | | | | | | | | | | | | | | | |  |  |  |  |  |
| --- | --- | --- | --- | --- | --- | --- | --- | --- | --- | --- | --- | --- | --- | --- | --- | --- | --- | --- | --- | --- | --- | --- | --- | --- | --- | --- | --- | --- | --- | --- | --- | --- |
|  |  | |  | |  | | | | | | | | | | | |  | | | | | | | | | | |  |  |  |  |  |
| **K for CA** | **Completed runs** | | **Aborted runs** | | **Sequence of plateau lengths** | | | | | | | | | | | | | | | | | | | | | | |  | |  |  |  |
| s2 | 50 | | 0 | | 12, 6, 2, 7, 4 | | | | | | | | | | | | | | | | | | | | |  | | |  | | | |
| 3 | 41 | | 9 | | 3, 2, 3, 2, 9, 3 | | | | | | | | | | | | | | | | | | | | |  | | |  | | | |
| 4 | 12 | | 38 | | 4, 2 | | | | | | | | | | | | | | | | | | | | |  | |  | | | |  |
| 5 | 0 | | 50 | | 0 | | | | | | | | | | | | | | | | | | | | |  | |  | | | |  |
| 6 | 0 | | 50 | | 0 | | | | | | | | | | | | | | | | | | | | |  | |  | | | |  |
| 7 | 0 | | 50 | | 0 | | | | | | | | | | | | | | | | | | | | |  | |  | | | |  |
| 8 | 0 | | 50 | | 0 | | | | | | | | | | | | | | | | | | | | |  | |  | | | |  |
| ** The sum of all plateau lengths is larger with k = 2 than with k = 3, thus given our small sample size* for K = 3 (below) *we proceeded with K = 2* to examiNE the geNE tic sources | | | | | | | | | | | | | | | | | | | | | | |  |  |  |  |  |  |  |  |  |  |
| **K = 3** | **SPECIMENS ALLOCATED** | | | |  | | | | | | | | |  | | | | | | |  |  |  |  |  |  |  |  |  |  |  |  |
|  | **ref1** | | | **ref2** | **ref3** | | | | | **Mean LLOD** | | | | | | | **2.37** | | | | | |  |  |  |  |  |  |  |  |  |  |
| **NE CA** | 7 | | | 9 | 8 | | | | | | | | |  | | | | | | |  |  |  |  |  |  |  |  |  |  |  |  |
|  |  | | |  |  | | | | | | | | |  | | | | | | |  |  |  |  |  |  |  |  |  |  |  |  |
| **K for TX** | Completed runs | | | Aborted runs | Sequence of plateau lengths | | | | | | | | | | | | | | | |  |  |  |  |  |  |  |  |  |  |  |  |
| 2 | 50 | | | 0 | 27, 3, 2, 6 | | | | | | | | | | | | | | | |  |  | |  |  |  |  |  |  |  |  |  |
| 3 | 46 | | | 4 | 4, 3, 3, 2 | | | | | | | | | | | | | | | |  |  | |  |  |  |  |  |  |  |  |  |
| 4 | 11 | | | 39 | 0 | | | | | | | | | | | | | | | |  |  | |  |  |  |  |  |  |  |  |  |
| **K = 2** | **SPECIMENS ALLOCATED** | | | |  | | | | | |  | | | | | | | | | |  |  |  |  |  |  |  |  |  |  |  |  |
|  | **ref1** | | | **ref2** |  | | | | **Mean LLOD** | | | | | | | | **3.01** | | | | | |  |  |  |  |  |  |  |  |  |  |
| **NE TX** | 11 | | | 11 |  | | | | | |  | | | | | | | | | |  |  |  |  |  |  |  |  |  |  |  |  |
|  |  | | |  |  | | | | | |  | | | | | | | | | |  |  |  |  |  |  |  |  |  |  |  |  |
| **K for UR** | Completed runs | | | Aborted runs | Sequence of plateau lengths | | | | | | | | | | | | | | | |  |  |  |  |  |  |  |  |  |  |  |  |
| 2 | 50 | | | 0 | | | 29, 2, 6, 2, 4, 4, 2 | | | | | | | | | | | | | | | |  | | | | | | | |  |  |
| 3 | 20 | | | 30 | | | 0 | | | | | | | | | | | | | |  |  | | | | | | | | |  |  |
| 4 | 0 | | | 50 | | | 0 | | | | | | | | | | | | | |  |  | | | | | | | | |  |  |
| **K = 2** | **SPECIMENS ALLOCATED** | | | | |  | | | | | | | | |  | | | | | |  |  |  |  |  |  |  |  |  |  |  |  |
|  | **ref1** | | | **ref2** | |  | | **Mean LLOD** | | | | | | | | | **3.84** | | | | | |  |  |  |  |  |  |  |  |  |  |
| **NE UR** | 16 | | | 15 | |  | | | | | | | | |  | | | | | | | |  | |  |  |  |  |  |  |  |  |
|  |  | | |  | |  | | | | | | | | |  | | | | | | | |  | |  |  |  |  |  |  |  |  |
|  |  | | |  | |  | | | | | | | | |  | | | | | | | |  | |  |  |  |  |  |  |  |  |
|  |  | | |  | |  | | | | | | | | |  | | | | | | | |  | |  |  |  |  |  |  |  |  |
|  |  | | |  | |  | | | | | | | | |  | | | | | | | |  | |  |  |  |  |  |  |  |  |
| **K for FL** | Completed runs | | | Aborted runs | | Sequence of plateau lengths | | | | | | | | | | | | | | | | |  | |  |  |  |  |  |  |  |  |
| 2 | 50 | | | 0 | | 12, 2, 11, 2, 6, 3, 2 | | | | | | | | | | | | | | | | |  | | | | | | | |  |  |
| 3 | 31 | | | 19 | | | | | | | 6, 3, 2, 2, 4 | | | | | | | | |  | | |  | | | | | | | |  |  |
| 4 | 8 | | | 42 | | | | | | | 2 | | | | | | | | |  | | |  | | | | | | | |  |  |
| **K =3** | **SPECIMENS ALLOCATED** | | | |  | | | | | | | | | |  | | | | | | | |  | |  |  |  |  |  |  |  |  |
|  | **ref1** | | | **ref2** | **ref3** | | | | | | **Mean LLOD** | | | | | | **3.00** | | | | | |  |  |  |  |  |  |  |  |  |  |
| **NE FL** | 6 | | | 6 | 9 | | | | | | | | | |  | | | | | | | |  | |  |  |  |  |  |  |  |  |
|  |  | | |  |  | | | | | | | | | |  | | | | | | | |  | |  |  |  |  |  |  |  |  |
| **K for SAG** | Completed runs | | | Aborted runs | Sequence of plateau lengths | | | | | | | | | | | | | | | | | |  | |  |  |  |  |  |  |  |  |
| 2 | 50 | | | 0 | 23, 27 | | | | | | | | | | | | | | | | | |  | |  | |  |  |  |  |  |  |
| 3 | 49 | | | 1 | 2, 2, 2, 2, 2 | | | | | | | | | | | | | | | | | |  | |  | |  |  |  |  |  |  |
| **K = 2** | **SPECIMENS ALLOCATED** | | | |  | | | | | | | | | |  | | | | | | | |  | |  |  |  |  |  |  |  |  |
|  | **ref1** | | | **ref2** |  | | | | | | | **Mean LLOD** | | | | | | | **2.65** | | | |  |  |  |  |  |  |  |  |  |  |
| **NE SAG** | **6** | | | **16** |  | | | | | | |  | | | | | | | | |  | |  |  |  |  |  |  |  |  |  |  |
|  |  | | |  |  | | | | | | | | | |  | | | | | |  | |  |  |  |  |  |  |  |  |  |  |
| **K for SAW** | Completed runs | | | Aborted runs | Sequence of plateau lengths | | | | | | | | | | | | | | | |  | |  |  |  |  |  |  |  |  |  |  |
| 2 | 50 | | | 0 | 4, 5, 8, 2, 3, 2, 4, 3, 2, 2, 2, 2 | | | | | | | | | | | | | | | |  | |  |  |  |  |  |  |  |  |  |  |
| 3 | 41 | | | 9 | 3, 4, 2, 2, 3, 2 | | | | | | | | | | | | | | | |  | |  | | | | | | | |  |  |
| 4 | 18 | | | 32 | 2 | | | | | | | | | | | | | | | |  | |  | | | | | | | |  |  |
| 5 | 1 | | | 49 | 0 | | | | | | | | | | | | | | | |  | |  | | | | | | | |  |  |
| **K = 2** | **SPECIMENS ALLOCATED** | | | |  | | | | | | | | | |  | | | | | |  | |  |  |  |  |  |  |  |  |  |  |
|  | **ref1** | | | **ref2** |  | | | | | | **Mean LLOD** | | | | | **3.09** | | | | | | |  |  |  |  |  |  |  |  |  |  |
| **NE SAW** | **12** | | | **11** |  | | | | | | |  | | | | | | | |  | | |  |  |  |  |  |  |  |  |  |  |
|  |  | | |  |  | | | | | | | | | |  | | | | |  | | |  |  |  |  |  |  |  |  |  |  |
| **K for SAK** | Completed runs | | | Aborted runs | Sequence of plateau lengths | | | | | | | | | | | | | | |  | | |  |  |  |  |  |  |  |  |  |  |
| 2 | 50 | | | 0 | 19, 2, 2, 5, 4, 3 | | | | | | | | | | | | | | |  | | |  |  |  |  |  |  |  |  |  |  |
| 3 | 50 | | | 0 | 6, 3, 2, 3, 2, 2, 2, 2 | | | | | | | | | | | | | | |  | | |  |  |  |  |  |  |  |  |  |  |
| 4 | 22 | | | 28 | 4, 2 | | | | | | | | | | | | | | |  | | |  | | | | | |  |  |  |  |
| **K = 3** | **SPECIMENS ALLOCATED** | | | |  | | | | | | | | | |  | | | | |  | | |  |  |  |  |  |  |  |  |  |  |
|  | **ref1** | | | **ref2** | **ref3** | | | | | | **MLLOD** | | | | | | | **3.16** | | | | |  |  |  |  |  |  |  |  |  |  |
| **NE SAK** | **9** | | | **6** | **7** | | | | | |  | | | | | | | | |  | | |  |  |  |  |  |  |  |  |  |  |
|  |  | | |  |  | | | | | | | | | |  | | | | |  | | |  |  |  |  |  |  |  |  |  |  |
| **K for UG** | Completed runs | | | Aborted runs | Sequence of plateau lengths | | | | | | | | | | | | | | | | | |  | |  |  |  |  |  |  |  |  |
| 2 | 50 | | | 0 | 4, 2, 2, 4, 2, 3, 4, 2, 2 | | | | | | | | | | | | | | |  | | |  |  |  |  |  |  |  |  |  |  |
| 3 | 50 | | | 0 | 2, 2, 4, 2, 2, 6, 2 | | | | | | | | | | | | | | |  | | |  |  |  |  |  |  |  |  |  |  |
| 4 | 21 | | | 29 | 0 | | | | | | | | | | | | | | |  | | |  | | | | | |  |  |  |  |
| 5 | 1 | | | 49 | 0 | | | | | | | | | | | | | | |  | | |  | | | | | |  |  |  |  |
| **K = 3** | **SPECIMENS ALLOCATED** | | | |  | | | | | | | |  | | | | | | |  | | |  |  |  |  |  |  |  |  |  |  |
|  | **ref1** | | | **ref2** | **ref3** | | | | | | | | **MLLOD** | | | | **2.38** | | | | | |  |  |  |  |  |  |  |  |  |  |
| **NE UG** | **11** | | | **9** | **6** | | | | | | | |  | | | | | | |  | | |  |  |  |  |  |  |  |  |  |  |
|  |  | | |  |  | | | | | | | |  | | | | | | |  | | |  |  |  |  |  |  |  |  |  |  |
| **K for SI** | Completed runs | | | Aborted runs | Sequence of plateau lengths | | | | | | | | | | | | | | | |  |  |  |  |  |  |  |  |  |  |  |  |
| 2 | 50 | | | 0 | 6, 5, 2, 3 | | | | | | | | | | | |  | | | |  |  |  |  |  |  |  |  |  |  |  |  |
| 3 | 50 | | | 0 | 5, 3, 2, 2, 2 | | | | | | | | | | | |  | | | |  |  |  |  |  |  |  |  |  |  |  |  |
| 4 | 45 | | | 5 | 2, 2 | | | | | | | | | | | |  | | | |  |  |  |  |  |  |  |  |  |  |  |  |
| 5 | 26 | | | 24 | 0 | | | | | | | | | | | |  | | | |  |  |  |  |  |  |  |  |  |  |  |  |
| **K = 2** | **SPECIMENS ALLOCATED** | | | |  | | | | | | |  | | | | |  | | | | | |  |  |  |  |  |  |  |  |  |  |
|  | **ref1** | | | **ref2** |  | | | | | | | **Mean LLOD** | | | | | **2.62** | | | | | |  |  |  |  |  |  |  |  |  |  |
| **NE SI** | **15** | | | **18** |  | | | | | | |  | | | | |  | | | | | |  |  |  |  |  |  |  |  |  |  |
|  |  | | |  |  | | | | | | |  | | | | |  | | | | | |  |  |  |  |  |  |  |  |  |  |
| **K for CH** | Completed runs | | | Aborted runs | Sequence of plateau lengths | | | | | | | | | | | | | | | |  |  |  |  |  |  |  |  |  |  |  |  |
| 2 | 50 | | | 0 | 12, 3, 2, 2 | | | | | | | | | | | |  | | | |  |  |  |  |  |  |  |  |  |  |  |  |
| 3 | 50 | | | 0 | 15, 8, 2 | | | | | | | | | | | |  | | | |  |  |  |  |  |  |  |  |  |  |  |  |
| 4 | 45 | | | 5 | 2, 3, 2 | | | | | | | | | | | |  | | | |  |  |  |  |  |  |  |  |  |  |  |  |
| **K = 3** | **SPECIMENS ALLOCATED** | | | |  | | | | | | |  | | | | |  | | | | | |  |  |  |  |  |  |  |  |  |  |
|  | **ref1** | **ref2** | | | **ref3** | | | | | | | **Mean LLOD** | | | | | **2.38** | | | | | |  |  |  |  |  |  |  |  |  |  |
| **NE CH** | **9** | **5** | | | **9** | | | | | | |  | | | | |  | | | | | |  |  |  |  |  |  |  |  |  |  |

**C. FLOCK ALLOCATION TABLES FOR EACH POPULATION**

| **AU analysis part 1** | | | | | | |  | | | | **MLLOD** | | | | | **P-value** | | | | | | |  |  |  |  |  |  |  |  |  |
| --- | --- | --- | --- | --- | --- | --- | --- | --- | --- | --- | --- | --- | --- | --- | --- | --- | --- | --- | --- | --- | --- | --- | --- | --- | --- | --- | --- | --- | --- | --- | --- |
|  | **ref1** | | | | | | **ref2** | | | | **2.91** | | | | | **<0.001** | | | | | | |  |  |  |  |  |  |  |  |  |
| **NE AU** | 1 | | | | | | 19 | | | |  | | | | |  | | | | | | |  |  |  |  |  |  |  |  |  |
| **NE FL** | 7 | | | | | | 14 | | | |  | | | | |  | | | | | | |  |  |  |  |  |  |  |  |  |
| **NE UR1** | **15** | | | | | | **1** | | | |  | | | | |  | | | | | | |  |  |  |  |  |  |  |  |  |
| **NE UR2** | 5 | | | | | | 10 | | | |  | | | | |  | | | | | | |  |  |  |  |  |  |  |  |  |
| **AU analysis part 2** | | | | | | |  | | | | **MLLOD** | | | | | **P-value** | | | | | | |  |  |  |  |  |  |  |  |  |
|  | **ref1** | | | | | | **ref2** | | | | **2.49** | | | | | **0.01** | | | | | | |  |  |  |  |  |  |  |  |  |
| **NE AU** | **8** | | | | | | **12** | | | |  | | | | |  | | | | | | |  |  |  |  |  |  |  |  |  |
| **NE FL** | **5** | | | | | | **16** | | | |  | | | | |  | | | | | | |  |  |  |  |  |  |  |  |  |
| **NE UR2** | **11** | | | | | | **4** | | | |  | | | | |  | | | | | | |  |  |  |  |  |  |  |  |  |
| **FL is the most likely genetic source to AU.** | | | | | | | | | | | | | | | |  | | | | | | |  |  |  |  |  |  |  |  |  |
| ***But, see DAPC at end of this appendix for further clarification*** | | | | | | | | | | | | | | | | | | | | | |  |  |  |  |  |  |  |  |  |  |
|  |  | | | | | |  | | | |  | | | | |  | | | | | | |  |  |  |  |  |  |  |  |  |
|  |  | | | | | |  | | | |  | | | | |  | | | | | | |  |  |  |  |  |  |  |  |  |
|  |  | | | | | |  | | | |  | | | | |  | | | | | | |  |  |  |  |  |  |  |  |  |
| **C. FLOCK ALLOCATION TABLES FOR EACH POPULATION, continued** | | | | | | | | | | | | | | | | | | | | | | | |  |  |  |  |  |  |  |  |
| **CH & SI analysis part 1** |  | | | | | |  | | | | **MLLOD** | | | | | **P-value** | | | | | | |  |  |  |  |  |  |  |  |  |
|  | **ref1** | | | | | | **ref2** | | | | **3.63** | | | | | **<0.001** | | | | | | |  |  |  |  |  |  |  |  |  |
| **NE CH** | **1** | | | | | | **22** | | | |  | | | | |  | | | | | | |  |  |  |  |  |  |  |  |  |
| **NE AU** | **16** | | | | | | **4** | | | |  | | | | |  | | | | | | |  |  |  |  |  |  |  |  |  |
| **NE FL** | **20** | | | | | | **1** | | | |  | | | | |  | | | | | | |  |  |  |  |  |  |  |  |  |
| **NE SAG** | **22** | | | | | | **0** | | | |  | | | | |  | | | | | | |  |  |  |  |  |  |  |  |  |
| **NE SAW** | **20** | | | | | | **3** | | | |  | | | | |  | | | | | | |  |  |  |  |  |  |  |  |  |
| **NE SAK** | **18** | | | | | | **4** | | | |  | | | | |  | | | | | | |  |  |  |  |  |  |  |  |  |
| **NE SI** | **1** | | | | | | **32** | | | |  | | | | |  | | | | | | |  |  |  |  |  |  |  |  |  |
| **NE UG** | **22** | | | | | | **4** | | | |  | | | | |  | | | | | | |  |  |  |  |  |  |  |  |  |
| **NE UR1** | **16** | | | | | | **0** | | | |  | | | | |  | | | | | | |  |  |  |  |  |  |  |  |  |
| **NE UR2** | **13** | | | | | | **2** | | | |  | | | | |  | | | | | | |  |  |  |  |  |  |  |  |  |
| **CH and SI are not sourced from any of the tested populations** | | | | | | | | | | | | | | | | | | | | | | | |  |  |  |  |  |  |  |  |
| **CH & SI analysis part 2** |  | | | | | |  | | | | **MLLOD** | | | | | **P-value** | | | | | | |  |  |  |  |  |  |  |  |  |
|  | **ref1** | | | | | | **ref2** | | | | **5.23** | | | | | **< 0.001** | | | | | | |  |  |  |  |  |  |  |  |  |
| **NE CH** | **23** | | | | | | **0** | | | |  | | | | |  | | | | | | |  |  |  |  |  |  |  |  |  |
| **NE SI** | **3** | | | | | | **30** | | | |  | | | | |  | | | | | | |  |  |  |  |  |  |  |  |  |
| **CH and SI are likely from separate population sources and are geNE tically distinct** | | | | | | | | | | | | | | | | | | | | | | |  |  |  |  |  |  |  |  |  |
| **CA1 analysis part 1** | | | | | | | | | | | **MLLOD** | | | | | **P-value** | | | | | | |  |  |  |  |  |  |  |  |  |
|  | **ref1** | | | | | | **ref2** | | | | **3.10** | | | | | **< 0.001** | | | | | | |  |  |  |  |  |  |  |  |  |
| **NE CA1** | 1 | | | | | | 10 | | | |  | | | | |  | | | | | | |  |  |  |  |  |  |  |  |  |
| **NE FL** | 2 | | | | | | 19 | | | |  | | | | |  | | | | | | |  |  |  |  |  |  |  |  |  |
| **NE TX1** | 0 | | | | | | 11 | | | |  | | | | |  | | | | | | |  |  |  |  |  |  |  |  |  |
| **NE TX2** | 0 | | | | | | 11 | | | |  | | | | |  | | | | | | |  |  |  |  |  |  |  |  |  |
| **NE UR1** | **15** | | | | | | **1** | | | |  | | | | |  | | | | | | |  |  |  |  |  |  |  |  |  |
| **NE UR2** | **10** | | | | | | **5** | | | |  | | | | |  | | | | | | |  |  |  |  |  |  |  |  |  |
| **CA1 analysis part 2** | | | | | | | | | | | **MLLOD** | | | | | **P-value** | | | | | | |  |  |  |  |  |  |  |  |  |
|  | **ref1** | | | | | | **ref2** | | | | **2.41** | | | | | **< 0.001** | | | | | | |  |  |  |  |  |  |  |  |  |
| **NE CA1** | **5** | | | | | | **6** | | | |  | | | | |  | | | | | | |  |  |  |  |  |  |  |  |  |
| **NE FL** | **10** | | | | | | **11** | | | |  | | | | |  | | | | | | |  |  |  |  |  |  |  |  |  |
| **NE TX1** | **11** | | | | | | **0** | | | |  | | | | |  | | | | | | |  |  |  |  |  |  |  |  |  |
| **NE TX2** | **7** | | | | | | **4** | | | |  | | | | |  | | | | | | |  |  |  |  |  |  |  |  |  |
|  | | | | | | | | | | |  | | | | |  | | | | | | |  |  |  |  |  |  |  |  |  |
|  | | | | | | | | | | |  | | | | |  | | | | | | |  |  |  |  |  |  |  |  |  |
| **C. FLOCK ALLOCATION TABLES FOR EACH POPULATION, continued** | | | | | | | | | | | | | | | | | | | | | | | |  |  |  |  |  |  |  |  |
| **CA1 analysis part 3** | | | | | | | | | | | **MLLOD** | | | | | **P-value** | | | | | | |  |  |  |  |  |  |  |  |  |
|  | **ref1** | | | | | | **ref2** | | | | **2.66** | | | | | **0.52** | | | | | | |  |  |  |  |  |  |  |  |  |
| **NE CA1** | 5 | | | | | | 6 | | | |  | | | | |  | | | | | | |  |  |  |  |  |  |  |  |  |
| **NE FL** | 9 | | | | | | 12 | | | |  | | | | |  | | | | | | |  |  |  |  |  |  |  |  |  |
| **NE TX2** | 7 | | | | | | 4 | | | |  | | | | |  | | | | | | |  |  |  |  |  |  |  |  |  |
| **Florida and TX2 are likely sources for CA1** | | | | | | | | | | | | | | | | | | | | | | |  |  |  |  |  |  |  |  |  |
| **CA2 analysis part 1** | | | | | | | | | | | **MLLOD** | | | | | **P-value** | | | | | | |  |  |  |  |  |  |  |  |  |
| Number of specimens among | **ref1** | | | | | | **ref2** | | | | **2.95** | | | | | **<0.001** | | | | | | |  |  |  |  |  |  |  |  |  |
| **NE CA2** | **13** | | | | | | **0** | | | |  | | | | |  | | | | | | |  |  |  |  |  |  |  |  |  |
| **NE FL** | 16 | | | | | | 5 | | | |  | | | | |  | | | | | | |  |  |  |  |  |  |  |  |  |
| **NE TX1** | 11 | | | | | | 0 | | | |  | | | | |  | | | | | | |  |  |  |  |  |  |  |  |  |
| **NE TX2** | 10 | | | | | | 1 | | | |  | | | | |  | | | | | | |  |  |  |  |  |  |  |  |  |
| **NE UR1** | **1** | | | | | | **15** | | | |  | | | | |  | | | | | | |  |  |  |  |  |  |  |  |  |
| **NE UR2** | **6** | | | | | | **9** | | | |  | | | | |  | | | | | | |  |  |  |  |  |  |  |  |  |
| **CA2 analysis part 2** | | | | | | | | | | | **MLLOD** | | | | | **P-VALUE** | | | | | | |  |  |  |  |  |  |  |  |  |
|  | **ref1** | | | | | | **ref2** | | | | **2.41** | | | | | **<0.001** | | | | | | |  |  |  |  |  |  |  |  |  |
| **NE CA2** | 12 | | | | | | 1 | | | |  | | | | |  | | | | | | |  |  |  |  |  |  |  |  |  |
| **NE FL** | **5** | | | | | | **16** | | | |  | | | | |  | | | | | | |  |  |  |  |  |  |  |  |  |
| **NE TX1** | 10 | | | | | | 1 | | | |  | | | | |  | | | | | | |  |  |  |  |  |  |  |  |  |
| **NE TX2** | **5** | | | | | | **6** | | | |  | | | | |  | | | | | | |  |  |  |  |  |  |  |  |  |
| **TX1 is the most likely source of CA2** | | | | | | | | | | | | | | | |  | | | | | | |  |  |  |  |  |  |  |  |  |
| **FL analysis part 1** | | | | | | |  | | | | **MLLOD** | | | | | **P-value** | | | | | | |  |  |  |  |  |  |  |  |  |
|  | **ref1** | | | | **ref2** | | | | | **3.07** | | | | | | | | | **<0.001** | | | | |  |  |  |  |  |  |  |  |
| **NE FL** | 19 | | | | 2 | | | | |  | | | | | | | | |  | | | | |  |  |  |  |  |  |  |  |
| **NE UR1** | **0** | | | | **16** | | | | |  | | | | | | | | |  | | | | |  |  |  |  |  |  |  |  |
| **NE UR2** | 8 | | | | 7 | | | | |  | | | | | | | | |  | | | | |  |  |  |  |  |  |  |  |
| **FL analysis part 2** | | | | | | |  | | | | **MLLOD** | | | | | **P-value** | | | | | | |  |  |  |  |  |  |  |  |  |
|  | **ref1** | | | | | | **ref2** | | | | **3.09** | | | | | **0.05** | | | | | | |  |  |  |  |  |  |  |  |  |
| **NE FL** | 15 | | | | | | 6 | | | |  | | | | |  | | | | | | |  |  |  |  |  |  |  |  |  |
| **NE UR2** | 4 | | | | | | 11 | | | |  | | | | |  | | | | | | |  |  |  |  |  |  |  |  |  |
| **UR2 is the most likely source of FL** | | | | | | | | | | | | | | | |  | | | | | | |  |  |  |  |  |  |  |  |  |
| **SAG analysis part 1** | | | | | | | | | | | **MLLOD** | | | | | **P-value** | | | | | | |  |  |  |  |  |  |  |  |  |
|  | **ref1** | | | | | | **ref2** | | | | **4.9** | | | | | **<0.001** | | | | | | |  |  |  |  |  |  |  |  |  |
| **NE SAG** | **22** | | | | | | **0** | | | |  | | | | |  | | | | | | |  |  |  |  |  |  |  |  |  |
| **NE AU** | 17 | | | | | | 3 | | | |  | | | | |  | | | | | | |  |  |  |  |  |  |  |  |  |
| **NE CH** | **0** | | | | | | **23** | | | |  | | | | |  | | | | | | |  |  |  |  |  |  |  |  |  |
| **NE FL** | 12 | | | | | | 9 | | | |  | | | | |  | | | | | | |  |  |  |  |  |  |  |  |  |
| **NE UR1** | 16 | | | | | | 0 | | | |  | | | | |  | | | | | | |  |  |  |  |  |  |  |  |  |
| **NE UR2** | 11 | | | | | | 4 | | | |  | | | | |  | | | | | | |  |  |  |  |  |  |  |  |  |
|  | | | | | | | | | | |  | | | | |  | | | | | | |  |  |  |  |  |  |  |  |  |
|  | | | | | | | | | | |  | | | | |  | | | | | | |  |  |  |  |  |  |  |  |  |
| **C. FLOCK ALLOCATION TABLES FOR EACH POPULATION, continued** | | | | | | | | | | | | | | | | | | | | | | | |  |  |  |  |  |  |  |  |
| **SAG analysis part 2** | | | | | | | | | | | **MLLOD** | | | | | **P-value** | | | | | | |  |  |  |  |  |  |  |  |  |
|  | **ref1** | | | | | | **ref2** | | | | **2.81** | | | | | **<0.001** | | | | | | |  |  |  |  |  |  |  |  |  |
| **NE SAG** | **22** | | | | | | **0** | | | |  | | | | |  | | | | | | |  |  |  |  |  |  |  |  |  |
| **NE AU** | 13 | | | | | | 7 | | | |  | | | | |  | | | | | | |  |  |  |  |  |  |  |  |  |
| **NE FL** | **6** | | | | | | **15** | | | |  | | | | |  | | | | | | |  |  |  |  |  |  |  |  |  |
| **NE UR1** | **2** | | | | | | **14** | | | |  | | | | |  | | | | | | |  |  |  |  |  |  |  |  |  |
| **NE UR2** | **6** | | | | | | **9** | | | |  | | | | |  | | | | | | |  |  |  |  |  |  |  |  |  |
| **AU is the most likely source of SAG** | | | | | | | | | | | | | | | |  | | | | | | |  |  |  |  |  |  |  |  |  |
| **SAW analysis part 1** | | | | | | | | | | | **MLLOD** | | | | | **P-value** | | | | | | |  |  |  |  |  |  |  |  |  |
|  | **ref1** | | | | | | **ref2** | | | | **4.35** | | | | | **<0.001** | | | | | | |  |  |  |  |  |  |  |  |  |
| **NE SAW** | **4** | | | | | | **19** | | | |  | | | | |  | | | | | | |  |  |  |  |  |  |  |  |  |
| **NE AU** | 1 | | | | | | 19 | | | |  | | | | |  | | | | | | |  |  |  |  |  |  |  |  |  |
| **NE CH** | **23** | | | | | | **0** | | | |  | | | | |  | | | | | | |  |  |  |  |  |  |  |  |  |
| **NE FL** | 2 | | | | | | 19 | | | |  | | | | |  | | | | | | |  |  |  |  |  |  |  |  |  |
| **NE UR1** | 0 | | | | | | 16 | | | |  | | | | |  | | | | | | |  |  |  |  |  |  |  |  |  |
| **NE UR2** | 3 | | | | | | 12 | | | |  | | | | |  | | | | | | |  |  |  |  |  |  |  |  |  |
| **SAW analysis part 2** | | | | | | | | | | | **MLLOD** | | | | | **P-value** | | | | | | |  |  |  |  |  |  |  |  |  |
|  | **ref1** | | | | | | **ref2** | | | | **2.87** | | | | | **<0.001** | | | | | | |  |  |  |  |  |  |  |  |  |
| **NE SAW** | **23** | | | | | | **0** | | | |  | | | | |  | | | | | | |  |  |  |  |  |  |  |  |  |
| **NE AU** | 16 | | | | | | 4 | | | |  | | | | |  | | | | | | |  |  |  |  |  |  |  |  |  |
| **NE FL** | 17 | | | | | | 4 | | | |  | | | | |  | | | | | | |  |  |  |  |  |  |  |  |  |
| **NE UR1** | **0** | | | | | | **16** | | | |  | | | | |  | | | | | | |  |  |  |  |  |  |  |  |  |
| **NE UR2** | **7** | | | | | | **8** | | | |  | | | | |  | | | | | | |  |  |  |  |  |  |  |  |  |
| **SAW analysis part 3** | | | | | | | | | | | **MLLOD** | | | | | **P-value** | | | | | | |  |  |  |  |  |  |  |  |  |
|  | **ref1** | | | | | | **ref2** | | | | **2.56** | | | | | **<0.001** | | | | | | |  |  |  |  |  |  |  |  |  |
| **NE SAW** | **8** | | | | | | **15** | | | |  | | | | |  | | | | | | |  |  |  |  |  |  |  |  |  |
| **NE AU** | **18** | | | | | | **2** | | | |  | | | | |  | | | | | | |  |  |  |  |  |  |  |  |  |
| **NE FL** | 7 | | | | | | 14 | | | |  | | | | |  | | | | | | |  |  |  |  |  |  |  |  |  |
| **FL is the most likely source of SAW** | | | | | | | | | | | | | | | |  | | | | | | |  |  |  |  |  |  |  |  |  |
| **SAK analysis part 1** | | | | | | | | | | | **MLLOD** | | | | | **P-value** | | | | | | |  |  |  |  |  |  |  |  |  |
|  | **ref1** | | | | | | **ref2** | | | | **4.35** | | | | | **< 0.001** | | | | | | |  |  |  |  |  |  |  |  |  |
| **NE SAK** | **4** | | | | | | **18** | | | |  | | | | |  | | | | | | |  |  |  |  |  |  |  |  |  |
| **NE AU** | 2 | | | | | | 18 | | | |  | | | | |  | | | | | | |  |  |  |  |  |  |  |  |  |
| **NE FL** | 2 | | | | | | 19 | | | |  | | | | |  | | | | | | |  |  |  |  |  |  |  |  |  |
| **NE UR1** | 0 | | | | | | 16 | | | |  | | | | |  | | | | | | |  |  |  |  |  |  |  |  |  |
| **NE UR2** | 2 | | | | | | 13 | | | |  | | | | |  | | | | | | |  |  |  |  |  |  |  |  |  |
| **NE CH** | **23** | | | | | | **0** | | | |  | | | | |  | | | | | | |  |  |  |  |  |  |  |  |  |
|  | | | | | | | | | | |  | | | | |  | | | | | | |  |  |  |  |  |  |  |  |  |
|  | | | | | | | | | | |  | | | | |  | | | | | | |  |  |  |  |  |  |  |  |  |
|  | | | | | | | | | | |  | | | | |  | | | | | | |  |  |  |  |  |  |  |  |  |
| **C. FLOCK ALLOCATION TABLES FOR EACH POPULATION CONTINUED** | | | | | | | | | | | | | | | | | | | | | | | |  | |  |  |  |  |  |  |
| **SAK analysis part 2** | | | | | | | | | | | **MLLOD** | | | | | **P-value** | | | | | | |  |  |  |  |  |  |  |  |  |
|  | **ref1** | | | | | | **ref2** | | | | **3.05** | | | | | **< 0.001** | | | | | | |  |  |  |  |  |  |  |  |  |
| **NE SAK** | **21** | | | | | | **1** | | | |  | | | | |  | | | | | | |  |  |  |  |  |  |  |  |  |
| **NE AU** | **15** | | | | | | **5** | | | |  | | | | |  | | | | | | |  |  |  |  |  |  |  |  |  |
| **NE FL** | **19** | | | | | | **2** | | | |  | | | | |  | | | | | | |  |  |  |  |  |  |  |  |  |
| **NE UR1** | **0** | | | | | | **16** | | | |  | | | | |  | | | | | | |  |  |  |  |  |  |  |  |  |
| **NE UR2** | **7** | | | | | | **8** | | | |  | | | | |  | | | | | | |  |  |  |  |  |  |  |  |  |
| **SAK analysis part 3** | | | | | | | | | | | **MLLOD** | | | | | **P-value** | | | | | | |  |  |  |  |  |  |  |  |  |
|  | **ref1** | | | | | | **ref2** | | | | **2.50** | | | | | **0.12** | | | | | | |  |  |  |  |  |  |  |  |  |
| **NE SAK** | **17** | | | | | | **5** | | | |  | | | | |  | | | | | | |  |  |  |  |  |  |  |  |  |
| **NE AU** | **10** | | | | | | **10** | | | |  | | | | |  | | | | | | |  |  |  |  |  |  |  |  |  |
| **NE FL** | **9** | | | | | | **12** | | | |  | | | | |  | | | | | | |  |  |  |  |  |  |  |  |  |
| **FL and AU are the most likely sources of SAK** | | | | | | | | | | | | | | | | | | | | | | |  |  |  |  |  |  |  |  |  |
| **TX1 analysis part 1** | | | | | | |  | | | | **MLLOD** | | | | | **P-value** | | | | | | |  |  |  |  |  |  |  |  |  |
|  | **ref1** | | | | | | **ref2** | | | | **3.35** | | | | | **<0.001** | | | | | | |  |  |  |  |  |  |  |  |  |
| **NE TX1** | **11** | | | | | | **0** | | | |  | | | | |  | | | | | | |  |  |  |  |  |  |  |  |  |
| **NE FL** | 18 | | | | | | 3 | | | |  | | | | |  | | | | | | |  |  |  |  |  |  |  |  |  |
| **NE UR1** | **1** | | | | | | **15** | | | |  | | | | |  | | | | | | |  |  |  |  |  |  |  |  |  |
| **NE UR2** | **6** | | | | | | **9** | | | |  | | | | |  | | | | | | |  |  |  |  |  |  |  |  |  |
| **TX1 analysis part 2** | | |  | | | | | | | | | | | **MLLOD** | | | **P-value** | | | |  | | | |  | | | |  |  |  |
|  | | | **ref1** | | | | | | **ref2** | | | | | | **2.99** | | **0.72** | | | | | | | | | | | | | |  |
| **NE TX1** | | | **7** | | | | | | **4** | | | | | |  | |  | | | | | | | | | | | | | |  |
| **NE FL** | | | 12 | | | | | | 9 | | | | | |  | |  | | | | | | | | | | | | | |  |
| **FL is the most likely source of TX1** | | |  | | | | | |  | | | | | | | | | | | | | | | |  |  |  |  |  |  |  |
| **TX2 analysis part 1** | | | | | | |  | | | | **MLLOD** | | | | | **P-value** | | | | | | |  |  |  |  |  |  |  |  |  |
|  | **ref1** | | | | | | **ref2** | | | | **3.13** | | | | | **<0.001** | | | | | | |  | | | | |  |  |  |  |
| **NE TX2** | **11** | | | | | | **0** | | | |  | | | | |  | | | | | | |  | | | | |  |  |  |  |
| **NE FL** | 19 | | | | | | 2 | | | |  | | | | |  | | | | | | |  | | | | |  |  |  |  |
| **NE UR1** | **1** | | | | | | **15** | | | |  | | | | |  | | | | | | |  | | | | |  |  |  |  |
| **NE UR2** | **11** | | | | | | **4** | | | |  | | | | |  | | | | | | |  | | | | |  |  |  |  |
| **TX2 analysis part 2** | | | | | | |  | | | | **MLLOD** | | | | | **P-value** | | | | | | |  | |  |  |  |  |  |  |  |
|  | **ref1** | | | | | | **ref2** | | | | **2.94** | | | | | **<0.01** | | | | | | |  | |  |  |  |  |  |  |  |
| **NE TX2** | **4** | | | | | | **7** | | | |  | | | | |  | | | | | | |  | |  |  |  |  |  |  |  |
| **NE FL** | **4** | | | | | | **17** | | | |  | | | | |  | | | | | | |  | |  |  |  |  |  |  |  |
| **NE UR2** | **12** | | | | | | **3** | | | |  | | | | |  | | | | | | |  | |  |  |  |  |  |  |  |
| **TX2 analysis part 2** | | | | | | |  | | | | **MLLOD** | | | | | **P-value** | | | | | | |  | |  |  |  |  |  |  |  |
|  | | | | **ref1** | | | | **ref2** | | | | **2.80** | | | | | | | | **0.71** | | | | |  |  |  |  |  |  |  |
| **NE TX2** | | | | **5** | | | | **6** | | | |  | | | | | | | |  | | | | |  |  |  |  |  |  |  |
| **NE FL** | | | | **11** | | | | **10** | | | |  | | | | | | | |  | | | | |  |  |  |  |  |  |  |
| **FL is the most likely source of TX2** | | | | | | |  | | | |  | | | | |  | | | | | | |  | |  |  |  |  |  |  |  |
|  | | | | | | |  | | | |  | | | | |  | | | | | | |  | |  |  |  |  |  |  |  |
|  | | | | | | |  | | | |  | | | | |  | | | | | | |  | |  |  |  |  |  |  |  |
| **UG analysis part 1** | | | | | | |  | | | | **MLLOD** | | | | | **P-value** | | | | | | |  | |  |  |  |  |  |  |  |
|  | **ref1** | | | | | | **ref2** | | | | **3.02** | | | | | **<0.001** | | | | | | |  | |  |  |  |  |  |  |  |
| **NE UG** | **26** | | | | | | **0** | | | |  | | | | |  | | | | | | |  | |  |  |  |  |  |  |  |
| **NE AU** | **15** | | | | | | **5** | | | |  | | | | |  | | | | | | |  | |  |  |  |  |  |  |  |
| **NE FL** | **19** | | | | | | **2** | | | |  | | | | |  | | | | | | |  | |  |  |  |  |  |  |  |
| **NE UR1** | **0** | | | | | | **16** | | | |  | | | | |  | | | | | | |  | |  |  |  |  |  |  |  |
| **NE UR2** | **6** | | | | | | **9** | | | |  | | | | |  | | | | | | |  | |  |  |  |  |  |  |  |
| **UG analysis part 2** | | | | | | |  | | | | **MLLOD** | | | | | **P-value** | | | | | | |  | |  |  |  |  |  |  |  |
|  | | **ref1** | | | | **ref2** | | | | | | | **2.43** | | | | | **0.03** | | | | | | |  | | | | |  |  |
| **NE UG** | | **6** | | | | **20** | | | | | | |  | | | | |  | | | | | | |  | | | | |  |  |
| **NE AU** | | **8** | | | | **12** | | | | | | |  | | | | |  | | | | | | |  | | | | |  |  |
| **NE FL** | | **13** | | | | **8** | | | | | | |  | | | | |  | | | | | | |  | | | | |  |  |
| **UG analysis part 3** | |  | | | | **MLLOD** | | | | | | | **P-value** | | | | |  | | | | | | |  | | | | |  |  |
|  | | **ref1** | | | | **ref2** | | | | | | | **2.52** | | | | | **0.02** | | | | | | |  | | | | |  |  |
| **NE UG** | | **18** | | | | **8** | | | | | | |  | | | | |  | | | | | | |  | | | | |  |  |
| **NE AU** | | **7** | | | | **13** | | | | | | |  | | | | |  | | | | | | |  | | | | |  |  |
| **AU is most likely source of UG** | |  | | | |  | | | | | | |  | | | | |  | | | | | | |  | | | | |  |  |
|  | | | | | | | | | | | | | | | | | | | | | | | | |  |  |  |  |  |  |  |
| **UG analysis part 2** | | | | | | |  | | | | **MLLOD** | | | | | **P-value** | | | | | | |  | |  |  |  |  |  |  |  |
|  | **ref1** | | | | | | **ref2** | | | | **2.43** | | | | | **0.03** | | | | | | |  | |  |  |  |  |  |  |  |
| **NE UG** | **6** | | | | | | **20** | | | |  | | | | |  | | | | | | |  | |  |  |  |  |  |  |  |
| **NE AU** | **8** | | | | | | **12** | | | |  | | | | |  | | | | | | |  | |  |  |  |  |  |  |  |
| **NE FL** | **13** | | | | | | **8** | | | |  | | | | |  | | | | | | |  | |  |  |  |  |  |  |  |
| **AU is the most likely source of UG** | | | | | | | | | | | | | | | |  | | | | | | |  | |  |  |  |  |  |  |  |

**Additional DAPC for clarification of FLOCK ALLOCATION TABLES**

Using the FLOCK allocation tables, we visualized the different potential clusters with DAPC to determine the potential genetic sources when FLOCK results were not clear.

| **AU analysis part 1** | |  | **MLLOD** | | **P-value** | |
| --- | --- | --- | --- | --- | --- | --- |
|  | **ref1** | **ref2** | **2.91** | | **<0.001** | |
| **NE AU** | 1 | 19 |  | |  | |
| **NE FL** | 7 | 14 |  | |  | |
| **NE UR1** | **15** | **1** |  | |  | |
| **NE UR2** | 5 | 10 |  | |  | |
| **AU analysis part 2** | |  | **MLLOD** | | **P-value** | |
|  | **ref1** | **ref2** | **2.49** | | **0.01** | |
| **NE AU** | **8** | **12** | |  | |  |
| **NE FL** | **5** | **16** | |  | |  |
| **NE UR2** | **11** | **4** | |  | |  |
| **FLOCK SUGGESTS AU AS COMPOSITE POPULATION, DAPC** (below) **HIGHLIGHTS THE GENETIC SOURCES FROM UR2 THAT HAVE GONE TO FL AND TO AU** | | | | | |  |

| **CA1 analysis part 1** | | | **MLLOD** | **P-value** |  |
| --- | --- | --- | --- | --- | --- |
|  | **ref1** | **ref2** | **3.10** | **< 0.001** |  |
| **NE CA1** | 1 | 10 |  |  |  |
| **NE FL** | 2 | 19 |  |  |  |
| **NE TX1** | 0 | 11 |  |  |  |
| **NE TX2** | 0 | 11 |  |  |  |
| **NE UR1** | **15** | **1** |  |  |  |
| **NE UR2** | **10** | **5** |  |  |  |
| **CA1 analysis part 2** | | | **MLLOD** | **P-value** |  |
|  | **ref1** | **ref2** | **2.41** | **< 0.001** |  |
| **NE CA1** | **5** | **6** |  |  |  |
| **NE FL** | **10** | **11** |  |  |  |
| **NE TX1** | **11** | **0** |  |  |  |
| **NE TX2** | **7** | **4** |  |  |  |
| **CA1 analysis part 3** | | | **MLLOD** | **P-value** |  |
|  | **ref1** | **ref2** | **2.66** | **0.52** |  |
| **NE CA1** | 5 | 6 |  |  |  |
| **NE FL** | 9 | 12 |  |  |  |
| **NE TX2** | 7 | 4 |  |  |  |
| **Florida and TX2 are likely sources for CA1** | | | | |  |
| **DAPC below confirms both are genetic sources to California CA1** | | | | |  |


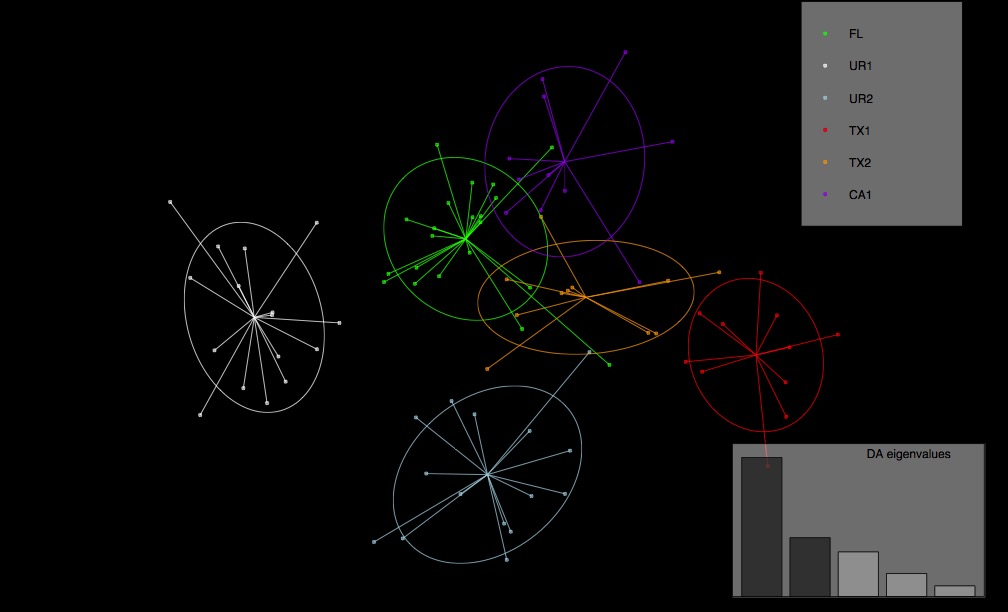

Supplement: Supplementary file 3 [file EVA-12-773-s003.docx]
