# Supplementary material for: Into the weeds: Matching importation history to genetic consequences and pathways in two widely used biological control agents
Source: Evol Appl. 2019 Jan 4;12(4):773–90. doi: 10.1111/eva.12755 (PMC6439500; doi:10.1111/eva.12755)
Supplement: Supplementary file 5 [file EVA-12-773-s005.docx]

**Analysis Methods**

In STRUCTURE, we ran an admixture model to compare and determine the number of distinct genetic clusters for each species across the collection sites from the native and introduced regions. We used the default settings in STRUCTURE to estimate the probability of assignment (Q) to each potential genetic cluster (K) with ten independent runs, each of 1 million generations with a burn-in period of 100,000 generations. We varied the number of genetic clusters (K) between 1 and 10 for *N. bruchi* and between 1 and 15 for *N. eichhorniae*. We used STRUCTURE HARVESTER (Earl & Vonholdt, 2012) to examine the structure result output files to determine the optimal K value based on the approach presented by (Evanno, Regnaut, & Goudet, 2005), and CLUMPAK (Cluster Markov Packager Across K) (Kopelman, Mayzel, Jakobsson, Rosenberg, & Mayrose, 2015) to visualize the plots from the optimal K.

**Results**

Analysis with the admixture model in STRUCTURE determined that the ‘true’ number of genetic clusters (populations) for *N. bruchi* ranged from K = 2 to K = 6 based on a peak ΔK of 19.21 and a mean Ln P(K) of -2495.86 at the plateau for respectively (Fig. S1a,b). Inspection of the admixture plots for K = 2 to K = 6 demonstrates genetic clustering of weevils from Texas, with similar genetic patterns to weevils from Uruguay, and differing from weevils from all other sites (Fig. S2).

For *N. eichhorniae,* analysis with STRUCTURE determined that the ‘true’ number of genetic clusters (populations) ranged from K = 2 based on ΔK which peaked at 1264.00 (Fig. S1c) to K = 6 where the mean Ln P(K) reached a plateau of -5794.48 (Fig. S1d). Analysis of the admixture plots indicate that weevils collected from China and Singapore are genetically distinct from weevils collected from the USA, South Africa, Australia and Uruguay, the latter representing the native range (Fig. S3). Inspection of the admixture plots for K = 3 to K = 6 indicates distinct genetic structure in the weevils collected from George in the Western Cape, South Africa (SAG) (Fig. S3). The STRUCTURE admixture plot for K = 6 additionally demonstrated clear genetic differences between the China and Singapore collection sites, as well as between Uruguay and Uganda collection sites (Fig. S3).

**References**

Earl, D. A., & Vonholdt, B. M. (2012). STRUCTURE HARVESTER: a website and program for visualizing STRUCTURE output and implementing the Evanno method. *Conservation Genetics Resources, 4*(2), 359-361. doi:10.1007/s12686-011-9548-7

Evanno, G., Regnaut, S., & Goudet, J. (2005). Detecting the number of clusters of individuals using the software STRUCTURE: a simulation study. *Molecular Ecology, 14*(8), 2611-2620. doi:10.1111/j.1365-294X.2005.02553.x

Kopelman, N. M., Mayzel, J., Jakobsson, M., Rosenberg, N. A., & Mayrose, I. (2015). Clumpak: a program for identifying clustering modes and packaging population structure inferences across K. *Mol Ecol Resour, 15*(5), 1179-1191. doi:10.1111/1755-0998.12387

**Fig. S1** STRUCTURE HARVESTER plots to find the Best K from the admixture model in STRUCTURE for *N. bruchi* with K = 1 - 10 clusters (A & B) and *N. eichhorniae* for K = 1-12 clusters (C & D). Using the method from Evanno et al. (2005): ΔK (A & C) and L(K) mean ± SD (C & D).

**Fig.** **S2** Genetic population structure of *Neochetina bruchi* based on 8 microsatellite markers as summarized from Clumpak based on results from an admixture model in STRUCTURE (with 10 runs per K-1 to K-10). Each plot visualizes major modes for K = 2 to K = 6, with the Best K = 2 based on ΔK, and the Best K = 6 based on the mean Ln P(K). Every vertical line represents 1 individual and the color demonstrates the proportion of each individual assigned to each of the potential clusters (2-6). Population abbreviations are defined in Table 1 of the main text.

**Fig. S3** Genetic population structure of *N. eichhorniae* based on 10 microsatellite markers as summarized from Clumpak based on results from an admixture model in STRUCTURE (with 10 runs per K-1 to K-12). Each plot visualizes major modes for K = 2 to K = 6, with the Best K = 2 based on ΔK, and the Best K = 6 based on the mean Ln P(K). Every vertical line represents 1 individual and the color demonstrates the proportion of each individual assigned to each of the potential clusters (2-4). Population abbreviations are defined in Table 1 of the main text.
